# The Burden of Antipsychotic-Induced Weight Gain and Metabolic Syndrome in Children

**DOI:** 10.3389/fpsyt.2021.623681

**Published:** 2021-03-12

**Authors:** Mark R. Libowitz, Erika L. Nurmi

**Affiliations:** Department of Psychiatry and Biobehavioral Sciences, Semel Institute for Neuroscience and Human Behavior, University of California, Los Angeles, Los Angeles, CA, United States

**Keywords:** child psychiatry, pediatrics, antipsychotics, antipsychotic-induced weight gain, adverse drug effects, metabolic syndrome

## Abstract

Antipsychotic medications are critical to child and adolescent psychiatry, from the stabilization of psychotic disorders like schizophrenia, bipolar disorder, and psychotic depression to behavioral treatment of autism spectrum disorder, tic disorders, and pediatric aggression. While effective, these medications carry serious risk of adverse events—most commonly, weight gain and cardiometabolic abnormalities. Negative metabolic consequences affect up to 60% of patients and present a major obstacle to long-term treatment. Since antipsychotics are often chronically prescribed beginning in childhood, cardiometabolic risk accumulates. An increased susceptibility to antipsychotic-induced weight gain (AIWG) has been repeatedly documented in children, particularly rapid weight gain. Associated cardiometabolic abnormalities include central obesity, insulin resistance, dyslipidemia, and systemic inflammation. Lifestyle interventions and medications such as metformin have been proposed to reduce risk but remain limited in efficacy. Furthermore, antipsychotic medications touted to be weight-neutral in adults can cause substantial weight gain in children. A better understanding of the biological underpinnings of AIWG could inform targeted and potentially more fruitful treatments; however, little is known about the underlying mechanism. As yet, modest genetic studies have nominated a few risk genes that explain only a small percentage of the risk. Recent investigations have begun to explore novel potential mechanisms of AIWG, including a role for gut microbiota and microbial metabolites. This article reviews the problem of AIWG and AP metabolic side effects in pediatric populations, proposed mechanisms underlying this serious side effect, and strategies to mitigate adverse impact. We suggest future directions for research efforts that may advance the field and lead to improved clinical interventions.

## Introduction

In the 1950s the first antipsychotic (AP) medication, chlorpromazine, became available for adults. These first-generation antipsychotic (FGAs) drugs made it possible to stabilize severe mental illness that previously required long-term institutionalization. While FGAs revolutionized the practice of psychiatry, serious motor adverse effects were common (1). This prompted the introduction of the first atypical antipsychotic (second-generation antipsychotic, SGA), clozapine, in 1990. SGA prescriptions soon dominated due to their reduced motor side effects and benefit in treating negative symptoms compared to FGAs (2, 3). SGAs also cause considerable morbidity, however, predominantly through antipsychotic-induced weight gain (AIWG) and metabolic dysfunction (1). Nevertheless, SGAs continue to represent the standard of care (93% of AP prescriptions as of 2008) (4), including for children and adolescents (age ≤ 19) (5).

The already dire prevalence of pediatric obesity and metabolic syndrome (6) is compounded by increasing global trends in pediatric AP prescribing, with children potentially accumulating risk over decades of pharmacotherapy. The mechanisms underlying these adverse effects are poorly understood, and consequently, few mitigating or alternative options are available to clinicians. This review will first outline AP exposure in the pediatric population, metabolic health consequences of pediatric AP treatment, and moderators of risk for adverse events (AEs). Next, an overview of proposed mechanistic pathways will be provided. Finally, we will summarize strategies to mitigate adverse impacts of these necessary therapeutics and synthesize a decision support algorithm for clinicians. We will conclude with future directions for research and treatment. An understanding of the biological underpinnings of metabolic AP effects is crucial to preventing negative physical and mental outcomes of youth in need of AP therapy and to designing new targeted treatments without burdensome side effects.

## Search Strategy, Selection Criteria, and Definition of Terms

In this narrative review, we attempted to limit bias and ensure comprehensiveness through broad search strategies. We searched PubMed, using the search terms “antipsychotic-induced weight gain,” “metabolic syndrome,” “cardiometabolic,” “pediatric OR adolescents OR child OR children,” “obesity,” “diabetes,” “second generation antipsychotics,” “neurohormone,” and “neuroendocrine.” Our search included articles published on PubMed through October 30th 2020. Publications were selected based on relevance, with priority given to publications from human research on antipsychotic-induced weight gain and metabolic effects from the past 10 years. We prioritized data from pediatric populations and provided adult data when this was lacking. With regard to treatment studies, we prioritized randomized controlled trials, systematic reviews and meta-analyses. With regard to genetic studies, we prioritized unbiased genome-wide studies. We also searched the reference list from articles and reviews identified by this strategy to select additional relevant titles. We supplemented the search with reviewer recommendations.

We use the following terms as defined by the American Association of Child and Adolescent Psychiatry (AACAP): “child” or “children” will refer to patients ages 5 to 12 years (or zero to 12 when specified), “adolescent(s)” to those between the ages of 13–17 years (inclusive) and “youth” to patients between ages 5 and 18 (7).

## Antipsychotic Exposure In Youth

Prescription rates of psychotropic medications vary by country, with US utilization exceeding that in Europe (8). A 2019 analysis of international data revealed that the highest prevalence estimates (~3%) for AP prescriptions in children and adolescents (age ≤ 19) occur in Taiwan and the US (9). A 2014 survey revealed that AP prescription rates are higher in publicly (2%) vs. privately (0.7%) insured US children and adolescents (0–19 years) (10). In the outpatient setting, the SGA most frequently prescribed to children aged 0-13 is risperidone (42.1%), followed by aripiprazole (28.0%), quetiapine (19.2%), and olanzapine (4.4%) (11).

Despite their name and primary use in treating psychosis, AP treatment is supported by evidence for a range of psychiatric disorders. Aggression, and not psychosis, is the most common symptom targeted by AP administration to youth (12–17). The National Ambulatory Medical Care Survey reported that from 2005–2009, APs were prescribed in 31.3% of outpatient visits for youth (age ≤ 20, *n* = 527) with mood disorders (11). The Food and Drug Administration has approved SGAs for use in children and adolescents with schizophrenia, type I and II bipolar disorder, Tourette disorder, and irritability related to autism spectrum disorder. Prescribing trends have shown an increase in SGA prescriptions for younger children (5, 18–24), including off-label use for childhood ADHD and depression for which AP therapy has limited evidence-base (22).

The typical reported duration of pediatric AP treatment varies. In a cohort of Australian patients <15 years of age prescribed APs (*n* = 901), the average duration of overall AP use was 2.4 years (25). The AP with the longest duration of use for this cohort was haloperidol followed by risperidone, chlorpromazine, olanzapine, quetiapine, aripiprazole, and lastly amisulpride. The most prescribed AP for this age group, risperidone, had a mean use of 2.25 years. In a Canadian Cohort of pediatric patients prescribed an SGA the most common diagnosis was ADHD, Mood Disorder, Conduct Disorder, or Psychotic Disorder. The median duration of risperidone, the most prescribed SGA for this cohort, was 179, 334, and 408 days for children aged 1–6 (*n* = 1,341), 7–12 (*n* = 17,356), and 13–18 (*n* = 32,604), respectively (26). A Medicaid-insured birth cohort examining trends in psychotropic prescription rates and medication use found that among 7-year-old children prescribed APs, 50.6% continued use for 6 months or more (27). Median duration of AP use increased with age, from 57 days in children aged 3 (*n* = 9) to 193 days in children aged 7 (*n* = 193). In this cohort only 15% of those prescribed an AP had a diagnosis of autism spectrum disorder, schizophrenia or bipolar disorder, revealing a trend of off-label AP prescriptions.

Weight gain is commonly reported by patients and physicians as an important factor in non-adherence (28–30). Discontinuation of pediatric AP treatment is common and determining the long-term severity of AEs after discontinuation is a concern (28–33). Both a naturalist study of (29) first-episode psychosis (*n* = 110, age range = 9–17, mean age = 15.3) treated with olanzapine, clozapine, or quetiapine and a controlled study (28) of early-onset schizophrenia spectrum disorder (*n* = 116, age range = 8–19) treated with olanzapine, risperidone, or molindone found that discontinuation of AP use within 12 months is the norm. In both studies, the main reasons cited by patients for discontinuation were insufficient response and AEs such as weight gain.

## Metabolic Effects of Antipsychotics In Youth

**Figure 1:**
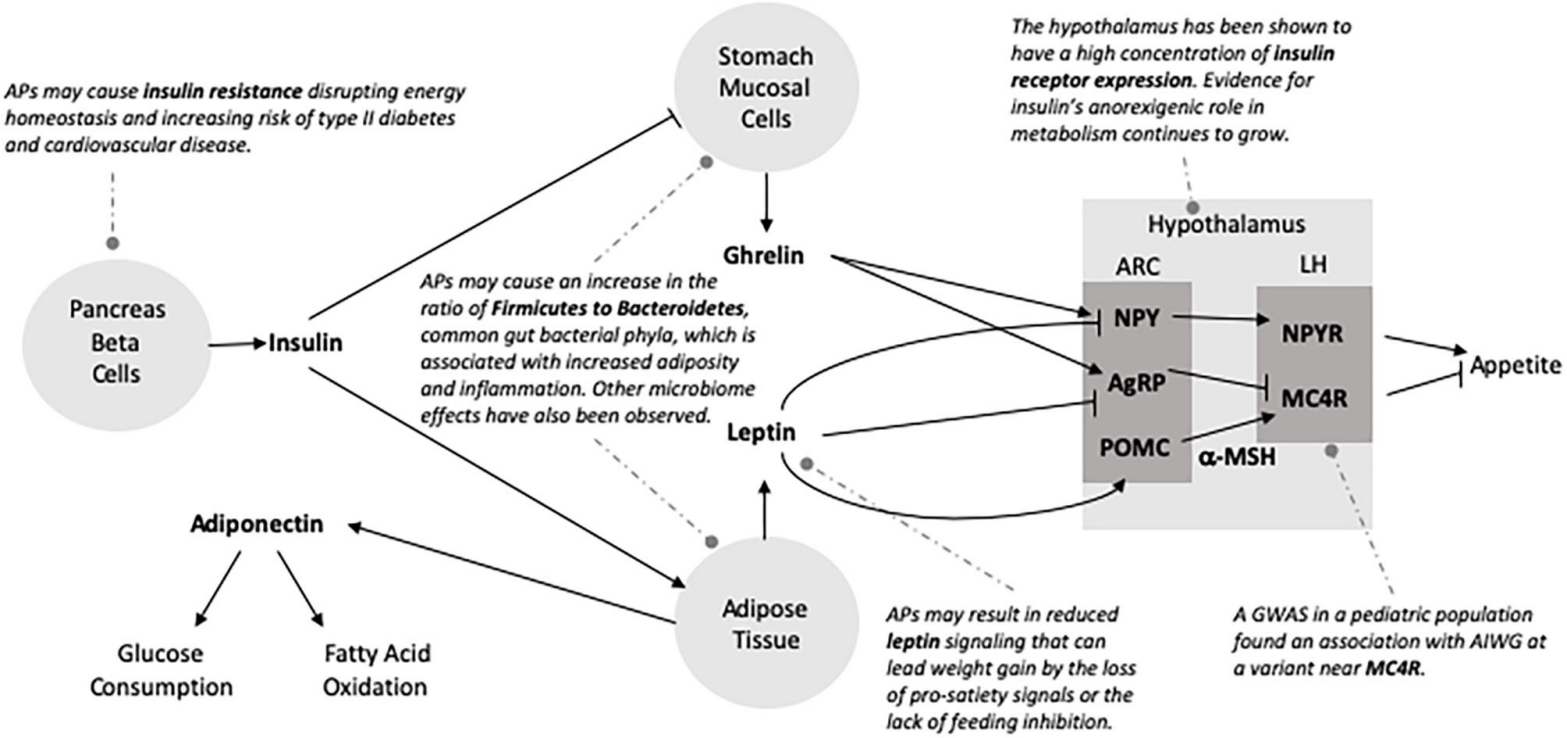
The associated burden of pediatric antipsychotic treatment. Antipsychotic use is associated with weight gain, increased fasting lipids (total cholesterol, LDL, and triglyceride levels), and impaired glucose tolerance in pediatric patients. Subsequently, this population is at an increased risk of obesity, metabolic syndrome and type 2 diabetes. Although pediatric patients are more vulnerable to these adverse side effects, they are less likely to have their metabolic parameters monitored during AP treatment.

Metabolic syndrome is a cluster of signs and symptoms, including insulin resistance, dyslipidemia, and hypertension, that increases subsequent risk of type 2 diabetes, heart disease, and stroke (Figure 1). APs can adversely impact metabolic function through direct effects on lipids and insulin sensitivity and indirect effects on these parameters as a result of AIWG and obesity (34–36). AIWG can be substantial, with average weight gain over a 12-month period measured at 5 kg, corresponding to a BMI increase by 1.5 in children and adolescents (mean age = 12, age range = 6–18, *n* = 200) (37). Importantly, AIWG increases the risk of obesity, which is predictive of both adult type 2 diabetes and adult metabolic syndrome (38). The International Childhood Cardiovascular Cohort Consortium consisting of 5,803 participants found a 2.4-fold increased risk for adult metabolic syndrome in children that are overweight with metabolic metrics above the 75th percentile from 5 years of age onward (39). Additionally, this study found increased risk (risk ratio = 2.6–4.1) for type 2 diabetes for children 8 years and older that were overweight and met 2 metabolic syndrome criteria.

Several studies have indicated that SGAs are associated with increased risk for metabolic symptoms. Results of a 2018 systematic review of 126 studies report AEs of APs in pediatric populations showed that compared to placebo, SGAs were associated with elevated triglyceride levels, weight gain, increased risk of type 2 diabetes, and unfavorable lipid changes (34). While this included only subjects under 18 years of age, the mean age across the studies was > 8 years, reducing its applicability to younger children. The SATIETY cohort (age range 4–19, mean age = 13.9, *n* = 205) study observed mean level increases in serum total cholesterol (15.6 mg/dL: both low-density lipoprotein, high density lipoprotein) and triglyceride (24.3 mg/dL) levels to increase with just a median of 10.8 weeks of exposure to SGAs (40). Further, patients developed dyslipidemia (17.1%), insulin resistance (8.6%), and metabolic syndrome (1.6%). In a mixed diagnosis comparison study of metabolic changes in adolescents (*N* = 179, age range = 12–18, mean age = 15.8) vs. adults (*N* = 4,280) receiving at least 24 weeks of olanzapine treatment, adolescents were found to have greater mean increases in fasting total cholesterol, LDL and triglyceride levels as compared to adults, while increases in fasting glucose levels were similar (41). Despite greater vulnerability, children and adolescents are less likely than adults to have their metabolic parameters monitored during AP treatment (37).

Type 2 diabetes has been implicated as a long-term AE of AP treatment in children and adolescents (42–45), as well as in adults (42, 43, 46). Studies examining this association are inconsistent. A retrospective study (47) evaluated South Carolina medical and pharmacy claims of children/adolescents receiving AP monotherapy (*n* = 30) or AP plus antidepressant treatment (*n* = 274) with type 2 (or misclassified type 1) diabetes did not attribute psychotropic medication as an explanatory factor of diabetes; however, causality cannot be inferred with a retrospective design and final group sizes are underpowered for most comparisons. Another retrospective cohort study (48) of outpatients (mean age 41.9, *SD* = 21.5) administered SGAs (*n* = 10,265), FGA (*n* = 4,607), antidepressants (*n* = 60,856), or antibiotics (*n* = 59,878) and a systematic review (*n* = 258,597 aged 0–5, *n* = 294,722 aged 6–11, and 331,339 aged 12–17) attributed risk of diabetes to non-specific factors given similar rates of diabetes with both APs and antidepressants (48, 49). A final retrospective national cohort study (age range = 10–18, 59.8% age 10–14, 40.2% age 15–18, *n* = 107,551) of youth receiving AP treatment reported higher risk of type 2 diabetes when antidepressants are used concomitantly with APs (45). Studies are also contradictory with regard to the relationship between risk for diabetes and age, with some reporting greater risk in older adolescents (44, 50) and others in younger patients (42). Although, there have been inconsistent findings for pediatric AP treatment and subsequent type 2 diabetes, data is strong enough to warrant regular physician monitoring of glucose levels (42, 44, 51).

## Risk for Antipsychotic-Related Metabolic Effects

Negative metabolic consequences and AIWG affect up to 60% of patients receiving APs, with the highest risk to children (40, 52–57). A multicenter naturalistic observational study (ETAPE) performed a 12-month follow up of AEs for 200 youth (mean age = 12, 92% prescribed SGAs) and found that the overall AE incidence rate was 11.52 AEs per person-years (37). For the AEs attributable to APs, 12.2% were related to metabolic or neuroendocrine parameters and included elevated cholesterol (>170 mg/dl) and triglycerides (≥100 mg/dl) (36.3%), hyperprolactinemia (>25 ng/ml) (38.5%), vitamin D deficiency (<30 ng/mL) (36.6%), hyperphagia (67.4%), and diabetes (7%). For the AEs recorded, more than half had incidence during the first 3 months of treatment. Moreover, children are more vulnerable to both the adverse physical and emotional effects of SGAs (24). As a result of this increased vulnerability, non-adherence in youth is prompted by changes in their physical appearance leading to body image issues (58) and negative peer perception (59).

Studies have shown that adverse health effects in youth increase with duration of treatment (60, 61) and that a younger age of AP use is associated with increased AIWG vulnerability (62), as well as AEs associated with obesity such as cardiovascular and metabolic complications (62–65). As research shows that most pediatric AIWG occurs within the first 12 weeks of administration (40, 65, 66), even relatively short-term treatment can result in considerable weight gain. First-episode psychosis is also a risk factor for greater weight gain, likely due to multiple factors (40, 67–69) such as younger age, lack of previous antipsychotic exposure, and less established participation in psychiatric treatment. Regular monitoring of adverse cardiometabolic effects for pediatric patients prescribed APs is standard of care (21, 70–77). Given accumulating risk over time and earlier age of initiation, longitudinal studies for pediatric AP use are highly warranted.

Propensity for weight gain and metabolic effects varies among AP agents. A 2018 network meta-analysis of 28 studies of pediatric AIWG (mean age = 14.41, 58% male) (78) found that molindone, lurasidone, and ziprasidone were relatively benign while clozapine, quetiapine, and olanzapine resulted in the greatest weight gain. Paralleling the adult literature, clozapine demonstrated both the greatest efficacy and side effect burden in youth. Importantly, medications touted to be weight neutral in adults behave differently in children. Aripiprazole, for example, has been noted to produce weight gain equivalent to or greater than risperidone in 2 pediatric studies (79, 80). The relationship of AP dose to AIWG remains unclear and may vary with time and across specific APs (81–83). Concomitant medications can also alter risk in either direction; for example, stimulants have been associated with attenuated (83) and mood stabilizers with compounded (84) risk for AIWG.

Diagnostic differences in weight gain have also been examined. A systematic review of children receiving AP treatment (*n* = 3,048) found that children diagnosed with autism spectrum disorder had higher propensity for weight gain, but this could be a result of younger age at treatment or lack of previous exposure to APs (66). Additionally, in a cohort of youth with schizophrenia or schizoaffective disorder, the Treatment of Early-Onset Schizophrenia Spectrum Disorders Study found that schizoaffective diagnosis predicted greater weight gain for risperidone prescribed youth (*n* = 119, age range = 8–19, *p* = 0.004) (85).

Considerable variability in weight gain and metabolic effects exists between individuals (86), though this variability is poorly understood. As previously discussed, young and antipsychotic-naïve patients are at particularly high risk, gaining 3–4-fold more weight irrespective of the specific AP (67). Few other patient-specific moderators of AIWG have been confirmed. Both higher and lower baseline BMI have been reported to predict AIWG in children (83, 87), which is complicated by confounding with age, AP exposure, and the expectation that extreme BMI values will regress toward the mean (83, 88). Reports of sex effects are also inconsistent, with studies claiming female (46, 89–91), male (63, 92), or equal (80) predominance of weight gain; though boys are prescribed APs more frequently than girls, paralleling male preponderance of many indications for AP administration (autism, Tourette, aggression). While AIWG and metabolic AEs appear to be a worldwide phenomenon, ethnicity and socioeconomic status may influence risk magnitude (92–94). APs are disproportionately prescribed more frequently to those in foster care (95) and to those with public insurance (96, 97).

Only a few studies have addressed the reversibility of AIWG (30–33). Two of these studies reported that AIWG in children and/or adolescents was reversible but are limited by small sample size (*n* = 14, mean age = 11.5; and *n* = 18, mean age = 9.68) and the inclusion of subjects who did not gain weight during AP treatment (31, 33). More moderately-sized studies showed contradicting results (30, 32). In a secondary analysis of AIWG in a pediatric placebo-controlled, cross-over study (age range = 5–17, mean age = 11.1, *n* = 527) of risperidone treatment of disruptive behavior disorders, those receiving placebo after 12 weeks of treatment underwent an average decrease of 0.2 kg (*SD* = 2.2 kg) over 6-months compared to an average of 3.2 kg (*SD* = 2.49 kg) gained during the treatment period (32). Upadhyay et al. (30) performed the most robust study to date, which showed only a fraction of weight gained during AP treatment is lost (average of +7.85 kg during treatment and−3.39 kg after 12-months discontinuation). This study limited its analysis to individuals who experienced any weight gain after AP treatment for a bipolar diagnosis before the age of 18 (*n* = 146). To date, it is unclear the extent to which pediatric AIWG is reversible. It is essential that future studies investigate the persistence of long-term metabolic outcomes in the context of AP discontinuation.

## Mechanisms Underlying Antipsychotic-Related Metabolic Syndrome and AIWG

Multiple mechanisms have been hypothesized to influence pediatric AIWG and metabolic effects of APs. It is likely that AEs are due to a combination of these mechanisms including AP influence on neurohormone receptor signaling and hormone mediation of APs, predisposition due to genetic risk factors, and AP effects on the gut microbiome. This review will summarize the main aspects of these mechanisms, as each has been thoroughly reviewed by other authors.

### Neurotransmitter Receptor Signaling

APs bind, with various affinities, to serotonin (5-HT), dopamine, histamine, adrenergic, and muscarinic cholinergic receptors (98–102). Several extensive reviews are available on neurotransmitter signaling as a potential mechanism in AIWG (Table 1) and metabolic effects of APs (101, 102, 112).

**Table 1:** Second generation antipsychotic neurotransmitter receptor binding profiles.

	**Second generation antipsychotic**								
**Receptor**	**ZPD**	**LRD**	**APZ**	**ASN**	**RSP**	**PPD**	**QTP**	**CLZ**	**OLZ**
5-HT_1A_	+++	+++	+++	+++	+	+	++	+	–
5-HT_1B_	+++		+	+++	++	++		+	+
5-HT_2A_	++++	+++	++	++++	++++	++++	++	++	+++
**5-HT**_**2C**_	**++++**	**+**	**++**	**++++**	**++**	**++**	**+**	**++**	**++**
5-HT_6_	++		+	++++	–	–	+	++	+++
D1	+	+		+++	+	+	+	+	++
**D2**	**+++**	**+++**	**+++**	**+++**	**+++**	**+++**	**+**	**+**	**++**
D3	+++		+++	++++	+++	+++	+	+	++
D4	++		+	+++	+++	+++		++	++
M1							+	+++	++
M3	–	–	–		–	–	+	++	++
**H1**	**++**	**–**	**++**	**+++**	**+++**	**++**	**+++**	**+++**	**+++**
H2				+++	+	+		+	++
H3									+
α1	++	++	++	+++	+++	+++	++	+++	++
**α2A**	**+**	**++**	**++**	**+++**	**++**	**+++**	**+**	**++**	**+**
α2B	++		++	++++	++	+++	+	++	++
α2C	++	+++	++	+++	+++	+++	++	++	++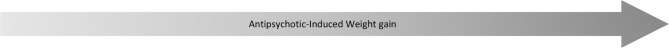									

*Inhibition constants (Ki) indicated as follows: ++++ (1 > Ki), +++ (1 < Ki < 10), ++ (10 < Ki < 100), + (100 < Ki < 1,000), –(1,000 < Ki), gray box indicates lack of data; data derived from (98–100, 103–111). Second Generation Antipsychotics are listed from left-to-right in order of increasing antipsychotic-induced weight gain; data derived from (78). This table does not provide an exhaustive receptor profile but focusses on receptors hypothesized to relate to metabolic effects. Those with the most evidence are bolded. ZPD, ziprasidone; LRD, lurasidone; APZ, aripiprazole; ASN, asenapine; RSP, risperidone; PDD, paliperidone; QTP, quetiapine; CLZ, clozapine; OLZ, olanzapine*.

#### Serotonin Signaling

Compared to FGAs, SGAs have greater affinity for 5-HT receptors than for dopamine receptors, conferring their reduced extrapyramidal side effects and superior efficacy in treating negative psychotic symptoms (2, 3) leading to preferential use in children (101, 113). Of the 5-HT receptors, SGA blockade of the serotonin 2C receptor (5- HT_2C_R) has been the most comprehensively studied. Rat models have shown reduced mRNA expression of 5- HT_2C_Rs in the hypothalamus, striatum, nucleus accumbens and amygdala with long-term clozapine administration (114). SGAs have shown high 5-HT receptor occupancy in neuroimaging studies (115), and there is longstanding evidence for the association of increased 5-HT levels and satiety (116). SGAs act to block 5-HT receptors including those in the hypothalamus, which play a central role in satiety signaling, and thus have been implicated as a candidate mechanism in AIWG (102, 112). Olanzapine and clozapine act as inverse agonists at the 5- HT_2C_R (117, 118) with lower affinity than aripiprazole, a partial agonist (119), but show greater AIWG (101). This evidence highlights the likely complex role of multiple mechanisms in AIWG.

### Histamine Signaling

Three histamine receptors are expressed in the brain (H1, H2, and H3). Histaminergic neurons originating in the posterior hypothalamus project throughout the brain and the H1 receptor, specifically, has been described to have a role in feeding behavior. In a study screening FGAs and SGAs, AP binding to the H1 receptor was most strongly associated with weight gain (99). In animal studies investigating clozapine and olanzapine, weight gain is associated with H1 receptor blockade, whereas agonist such as betahistine reduced olanzapine-induced weight gain (120, 121) Further, H1 antagonism by olanzapine and clozapine is proportional to the activation of AMP-activated protein kinase (AMPK) (122, 123), which has been shown to reduce the anorexigenic effects of leptin (124). These associations should be interpreted with caution, as both clozapine and olanzapine have high affinity for multiple receptors.

#### Other Neurotransmitter Signaling

SGAs result in lower occupancy of dopamine D2 receptors (D2R) as compared to FGAs but still bind these receptors as antagonists (98–100, 125). AP administration results in decreased striatal D2R availability (102, 126) and it has been hypothesized that overeating compensates for reduction of D2-regulated reward circuits resulting in increased caloric intake (126–128). SGAs also act as antagonists at D4 and agonists at D1 receptors (98). Many APs have strong affinity for adrenergic receptors which have been more heavily implicated in metabolic effects of APs due to α1 and α2 receptor association with glucose control (101) and the ratio of α2 to β3 in adipocyte hyperplasia (129, 130). SGAs have high affinity for cholinergic muscarinic receptors and blockade of M3 has been proposed to disrupt insulin homeostasis (131), but there is lack of data for a role in AIWG. Of the early hypotheses related to AP effects on neurohormone signaling based on genetic candidate gene data, none of the genetic associations with AIWG have been strengthened by concurrent evidence from unbiased genome studies.

### Neuroendocrine Signaling

**Figure 2:**
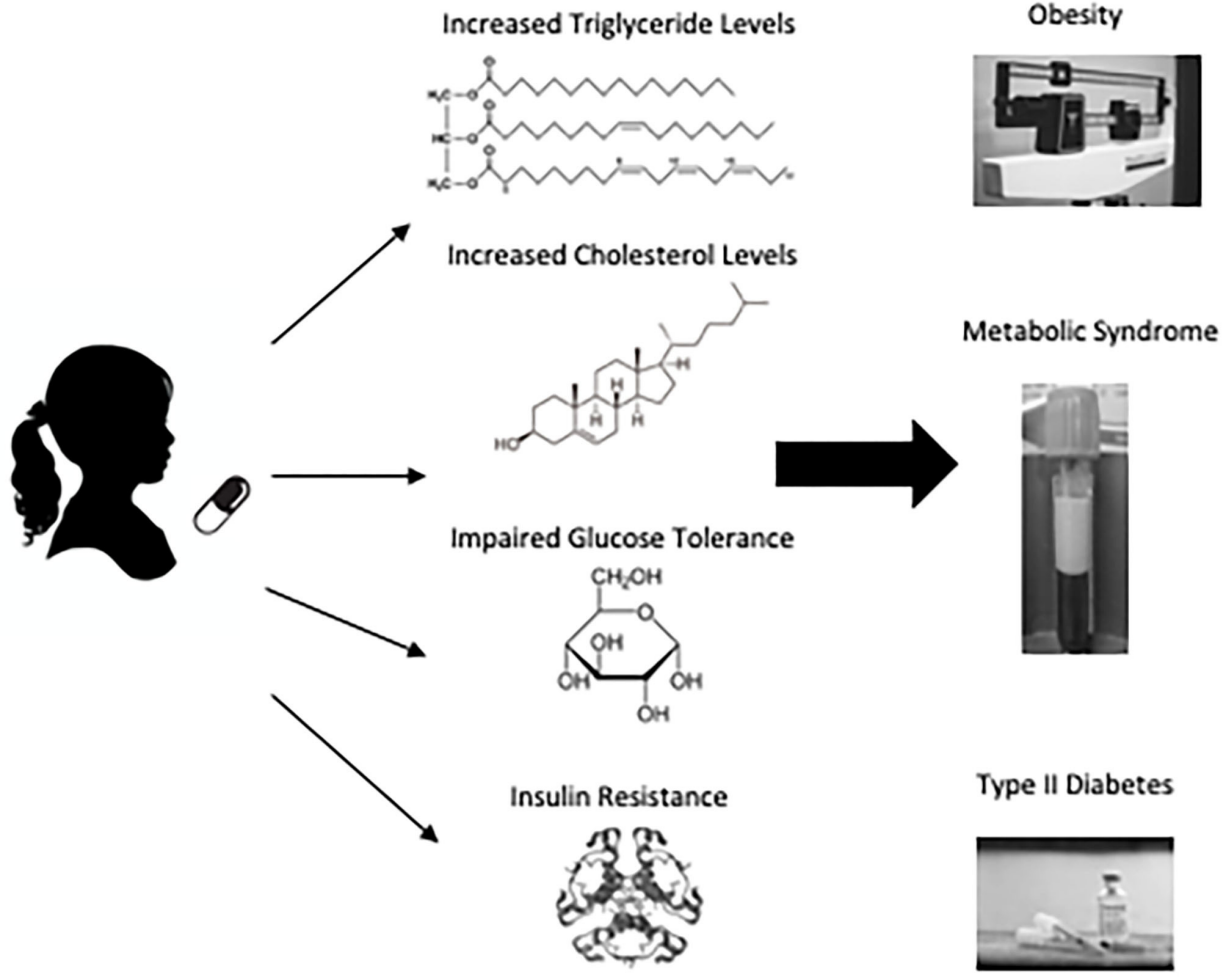
Possible mechanisms of antipsychotic adverse metabolic effects. The energy homeostasis pathway is complex and provides numerous possible mechanisms that might explain AP-related weight gain and metabolic effects. Arrow at end of line indicates activation of pathway and perpendicular bar denotes inhibition. Neuroendocrine signaling molecules are bolded. Potential AP inputs are italicized and represented by dotted lines. ARC, arcuate nucleus; LH, lateral hypothalamus; NPY, neuropeptide Y; AgRP, agouti-related peptide; POMC, pro-opiomelanocortin; NPYR, neuropeptide Y receptor; MC4R, Melanocortin 4 Receptor; α-MSH, alpha-Melanocyte-stimulating hormone.

Metabolic effects associated with APs could result from direct changes to neuroendocrine signaling or occur secondary to weight gain. AP effects on adiponectin, ghrelin, insulin, and leptin (Figure 2) have been examined as potential mediators of AP-related changes in energy homeostasis (132). These signaling molecules impact various levels of energy balance including appetite and feeding, energy expenditure and metabolic rate. Insulin and leptin modulate expression of neuropeptides in the hypothalamus, which regulate feeding behavior and are considered the most important agents in regulating weight gain and energy homeostasis (133).

#### Leptin Signaling

A review published by Endomba et al. (134) provides an excellent overview of the potential influence of APs on leptin metabolism. In brief, leptin acts on neurons of the lateral arcuate nucleus within the hypothalamus inhibiting the expression of neuropeptide Y (NPY) and agouti-related peptide (AgRP) and stimulating proopiomelanocortin (POMC) (135–137). POMC is modified to α-melanocyte-stimulating hormone, which can then stimulate melanocortin receptor 3 (MC3R) and 4 (MC4R), suppressing food intake (137). Mouse models have shown that structural MC4R alterations and decreased MC3R expression are associated with leptin resistance and obesity (137, 138). In addition to NPY and AgRP, leptin also acts to inhibit neurons in the ventromedial arcuate nucleus that express gamma-amino butyric acid (GABA), which induce feeding (139). Thereby, reduced leptin signaling can lead to obesity by the loss of pro-satiety signals or the lack of feeding inhibition (135). Yet, the overall effects of APs on leptin remain unclear despite extensive investigation. For example, increased leptin levels have been associated with SGA treatment in patients with schizophrenia (140). It has been proposed that leptin metabolism can be affected by AP treatment independent of AIWG (141–146), or conversely, only secondary to AIWG rather than by direct effects (147–152).

### Insulin Signaling

APs have been shown to increase insulin resistance (40). Insulin is produced in the pancreas by beta cells and binds to receptors in the arcuate nucleus aiding in energy homeostasis. Insulin resistance occurs when the activity of insulin is blunted in liver, muscle and adipose tissue and is linked to intra-abdominal fat (153). Childhood-onset insulin resistance increases risk for type 2 diabetes and cardiovascular disease (154, 155). AP actions on histamine and muscarinic receptors have been shown to reduce acetylcholine-induced insulin secretion (156, 157) and result in the failure of leptin signaling (158), which may contribute to insulin resistance. More recently, the hypothalamus has been shown to have a high concentration of insulin receptor expression and insulin concentration *in vitro* (159). In post-mortem studies receptor expression has been revealed to be greatest in the cerebellum and hypothalamus (160). Insulin has been shown to play an anorexigenic role in the brain and brain imaging studies have revealed a reduced neural response in patients with obesity upon exogenous insulin administration (160, 161). Insulin produced by the pancreas enters the brain via the bloodstream through an insulin-receptor mediated pathway initiating the phosphoinositide 3-kinase pathway that plays a crucial role in controlling metabolism (160). Additionally, insulin is involved in the stimulation of leptin secretion (162), and reciprocally, leptin plays a role in the regulation of circulating insulin (153). Human imaging studies have revealed that central insulin modulates the activity of mesocorticolimbic dopaminergic circuitry (163–165). In psychiatric patients, the development of type 2 diabetes and adverse metabolic effects may be facilitated by insulin resistance in the brain (166). Figure 2 depicts aspects of insulin's role in the CNS. For a more comprehensive review of the role insulin plays in the central nervous system as it relates to metabolism see Kullmann et al. (160).

#### Other Neurohormone Signaling

Adiponectin is secreted by adipose tissue and increases fatty acid oxidation and glucose uptake in muscle thereby contributing to weight regulation (167). Decreased adiponectin serum levels have been associated with insulin resistance, dyslipidemia, obesity and type 2 diabetes (168, 169). Meta-analysis found that SGA-treatment in patients with schizophrenia was associated with decreased levels of adiponectin (170). Ghrelin is a peptide hormone secreted in the stomach that has orexigenic effects by increasing food intake and fat deposits. Insulin has been evidenced to decrease ghrelin levels (171). Paradoxically, meta-analysis has provided some evidence that olanzapine-associated weight gain is associated with reduced ghrelin levels but associations with increased ghrelin in the context of SGA treatment has been reported as well (172–174). The circadian and immune regulator, melatonin, also plays a role in energy metabolism (175). Increased weight and visceral adiposity in olanzapine-treated rats is inversely proportional to nocturnal circulating melatonin (176) and daily melatonin supplementation ameliorates this effect (176, 177). More longitudinal studies with greater sample size are necessary to determine the relationship of AP treatment and these neurohormones.

### Genetic Predisposition

Genetic risk factors likely play an important role in the extent to which an individual experiences AIWG. In a study of monozygotic twins and siblings receiving SGAs, the influence of genetic factors on AIWG was reported to be between 60 and 80% (178). Candidate gene studies have focused on neurotransmitter receptors including 5-HT_2C_R *(HTR2C)* (142, 148, 179–183), D2R *(DRD2)* (184–188), α2-adrenergic receptor *(ADRA2A)* (189–193) and cannabinoid 1 receptor *(CNR1)* (194–197); energy balance regulators including *MC4R* (198–201), leptin *(LEP)* (145, 202–205) and transcription factor *SREBP* (206–209); and growth and synaptic genes like brain-derived neurotrophic factor *(BDNF)* (210–215) and synaptosomal associated protein *SNAP25* (216). For a comprehensive review of the candidate gene studies associated with AIWG and metabolic effects of APs see Li et al. (217).

Candidate gene studies have exploratory value but are weakened as they are based on functional hypotheses with inherent bias (218–220). The genetics field has agreed that candidate gene studies should be interpreted with extreme caution (221). Large unbiased genome-wide studies, such as genome wide association studies (GWAS), are thus necessary to reveal replicable genetic risk factors for complex phenotypes. The common variation model postulates that susceptibility to complex disease is driven by a combination of common alleles that each carry a small disease risk, such as can be revealed in large-scale GWAS analyses. For example, genetic risk for AIWG at *MC4R* was first identified in the only GWAS study of weight gain in pediatric patients (age ≤ 19, *n* = 139) taking quetiapine, risperidone, olanzapine, or aripiprazole for any diagnosis (222). This GWAS study revealed a single nucleotide polymorphism (SNP) at locus rs489693 located downstream from the *MC4R* gene. Independent studies investigating SNPs near *MC4R* subsequently replicated this finding (198–201). MC4R plays a central role in energy balance. As described above, leptin stimulates hypothalamic POMC neurons, resulting in the production of α-MSH, which in turn stimulates anorexigenic effects by binding MC4R and inhibiting AgRP, an MC4R antagonist (102). 5-HT_2C_R has upstream inputs to this pathway (223), and BDNF has been suggested to have effects downstream of MC4R, as its infusion in MC4R-deficient mouse models reduces food intake (224).

Several GWAS of AIWG have been performed in adult samples receiving APs to treat schizophrenia. Two studies utilized data from the Clinical Antipsychotic Trials of Intervention Effectiveness (CATIE) (225), both failing to detect genome-wide significant signals. The first also reported trends in SNPs upstream of opioid growth factor receptor *OGFRL1* (226), and the second highlighted enrichment of nominally associated SNPs in energy balance pathway genes by hypothesis-driven pathway enrichment analysis (227). A SNP (rs10977144) located in the protein tyrosine phosphatase, receptor type D gene *(PTPRD)* (228) was associated with AIWG in a GWAS of Chinese patients with schizophrenia (*n* = 524, mean age = 26.4). *PTPRD* deficient mice were shown to have insufficient weight gain postnatally due to feeding difficulties, arguing for a role in energy balance (229). A replication GWAS of European and African ancestry (*n* = 201, mean age = 37) treated primarily with clozapine or olanzapine did not confirm the lead SNP, which may be explained by unmatched ancestry, but notably did detect nominal significance at other SNPs within the *PTPRD* gene (230). This same GWAS found only marginal association (*p* <0.05) at a SNP located near the *MC4R* gene. In an additional study of this cohort, a SNP (rs1525085) in the lipid biosynthesis gene, diacylglycerol kinase beta *(DGKB)*, was found to be significantly associated with AIWG (231). The study notes that *DGKB* variants have been associated with insulin clearance (232) and, by interaction with insulin secretion, increased risk for type 2 diabetes (233). When limiting analyses to the European subset, a SNP (rs62097526) downstream of *CIDEA*, a regulator of lipolysis and thermogenesis in mice, was nominally associated with AIWG (231). *CIDEA* variants have also been shown to associate with metabolic syndrome in Swedish, Japanese and Chinese population cohorts as well as obesity risk in a Han-Chinese cohort (234, 235). The most recent GWAS (236) examining AIWG analyzed 339 subjects (Age Range = 15-45, mean age = 26.4) with first-episode psychosis derived from The Optimization of Treatment and Management of Schizophrenia in Europe (OPTiMiSE) (237) cohort. This study identified the intergenic SNP rs78310016 near *SEPP1*, a hepatic protein involved in selenium transport, and growth hormone receptor (GHR) that was significantly associated with AIWG but not replicated in follow-up analyses. Providing face validity to this finding, SEPP1 and GHR have been implicated in metabolic phenotypes (238–243). Another possible functional link was identified by *in silico* analysis, which predicted a chromatin interaction of the lead SNP with the HMG-CoA synthase 1 gene *(HMGCS1)*. HMGCS1 is highly expressed in the brain and liver and involved in the regulation of cholesterol biosynthesis (244).

Overall, while candidate gene studies may provide mechanistic clues in pediatric AIWG and metabolic effects of APs, large GWAS studies are required to definitively identify risk genes. Currently, a few GWAS samples exist but are underpowered and difficult to harmonize given different ages and ancestries. Moreover, only one study examined pediatric participants (222). Existing studies are derived almost entirely from treatment studies of schizophrenia, which due to time and expense are generally limited to hundreds of patients. Sample sizes consisting of thousands of patients will be necessary to comprehensively capture genetic contributions of common variants to AIWG. Data from electronic medical records, national registries, and commercial genetic profile industries may represent fruitful avenues for future study.

### The Microbiome

SGAs have been associated with perturbations of the gut microflora that may contribute to weight gain (245–248). The mechanism underlying alterations in the microbiome during SGA treatment and the link between these changes and weight gain are only beginning to be explored.

Several distinct observations have emerged from recent work including evidence that microbiome changes are necessary for AIWG rather than the reverse. AP administration in rodents and humans (249–252) results in an increase in the ratio of *Firmicutes* to *Bacteroidetes* (F:B), two common bacterial phyla. This observation parallels findings in obesity; however, systematic reviews in pediatric (253) and adult (254) populations identify inconsistencies. Preclinical studies have demonstrated that gut bacteria are necessary for AIWG (245, 249–251). In fact, AIWG is absent in germ-free mice but can be induced by microbiome transplant (251). Similar F:B changes, increased adiposity, and inflammation were reported in olanzapine-treated rats (249). In a follow-up study, these effects could be prevented by co-administration of an antibiotic cocktail that effectively sterilized the gut (250). Similarly, mice receiving risperidone developed AIWG, an effect that was mediated by decreased energy expenditure and transferrable by fecal transplant (245). Small studies investigating risperidone treated children substantiated these preclinical findings. Risperidone treated children (*n* = 18, age range = 9–15, mean age = 12.2) were observed, cross-sectionally, to have an elevated F:B ratio and a host of differences in the metabolic potential of the gut microbiota (252). In an independent longitudinal study published in the same report, children (*n* = 5, age range = 9–13, mean age = 11.7) were enrolled within days (mean = 3.2, *SD* = 5.2) of starting risperidone (252). Within 1–3 months of risperidone initiation, the F:B ratio had begun to increase, appearing to plateau by about 5–6 months. Importantly, the F:B ratio was positively correlated with the magnitude of AIWG. An overall increase in putative “obese gut microbiota” was seen for these adolescents, and interestingly an enrichment in microbiota genes related to serotonin signaling and short chain fatty acid metabolism was reported.

Additional microbiota alterations associated with AP treatment include changes in *Actinobacteria*, although both increases (245, 255) and decreases (249) in the phylum has been reported. Risperidone-treated mice display increased abundance of the *Erysipelotrichaceae* (256) family and *Mollicutes* (257) class but decreases in *Alistipes* and *Akkermansia* species, which are considered lean gut microbiota (258, 259). Reduced diversity and stability of the microbiome of children compared to adults could explain the increased sensitivity to AIWG seen in youth (65, 260, 261). Lower microbiota species diversity is associated with obesity (255) dysbiosis in youth may mediate subsequent adult obesity (65). Childhood microbiota composition has also been shown to be instrumental for brain and immune system development and function (262). Therefore, given their lower baseline microbiota population diversity, long-term disturbance of brain and immune system development as a result of SGA-associated with changes in gut microbiota should be examined. Lastly, a study that investigated 117 adult patients with bipolar disorder (*n* = 49 treated with an AP, *n* = 68 non-treated) a greater AP-related reduction in bacterial diversity was seen in females vs. males treated with an AP (263), suggesting that sex differences must also be considered.

## Pharmacological and Lifestyle Interventions to Address Metabolic AEs of APs

Current strategies to prevent or treat AIWG and the metabolic effects associated with AP use are inadequate. Unfortunately, the two most effective SGAs on the market, clozapine and olanzapine, also have the highest reported AIWG (78). Clinicians should understand and weigh the benefits and risks of SGA treatment for each individual patient. In some situations, it may be possible to avoid APs if behavioral strategies or medications with less AEs are implemented. Nevertheless, in children with acute psychosis or mania, there are often few other appropriate options. In cases where benefit outweighs risk, it is crucial to warn patients and families about AIWG and metabolic effects and useful to discuss strategies to reduce harm. The most conservative approaches to metabolic side effects, which can also be safely employed for prevention, are non-pharmacologic interventions, which include lifestyle modification and dietary supplementation, including pre- and probiotic supplements. When these approaches are insufficient, switch to an AP with less propensity for weight gain may be warranted or adjunctive medications may be added to manage weight gain. The most effective pharmacologic interventions, however, will likely be supported by a healthy lifestyle.

### Non-pharmacological Treatments

There is a paucity of data examining lifestyle interventions for AIWG in children (264). A 52-week study found that standard (*n* = 102) or intense behavioral weight interventions (n = 103) did not reduce AEs of APs for adolescents (age range = 13–17, mean age = 15.8) with schizophrenia or bipolar I disorder receiving olanzapine (265). A 16-week intensive weekly family-based behavioral weight loss intervention in AP-treated youth (*n* = 19, mean age = 13.35), compared to treatment as usual, resulted in decreased adiposity (*p* = 0.01) and hepatic fat (*p* = 0.04) that could support beneficial impacts on AIWG with long-term behavioral intervention (266). There is a need for large-scale pediatric studies to determine the most effective lifestyle interventions for weight gain and metabolic symptoms. As childhood obesity continues to present a major health challenge, a robust research literature exists on effective lifestyle interventions for obese youth. A comprehensive review of such integrative approaches was recently published (267) describing potential dietary, physical activity, sleep, and stress management interventions. Nutritional supplements with data in pediatric obesity, though not yet tested in the context of AP treatment, are also reviewed. The interventions with the best support in reducing childhood obesity and subsequent metabolic symptoms include an increased level of physical activity, improved sleep, and a diet consisting of fruits, vegetables, whole grains, and fish oil supplementation (267). Generalizing from healthy populations must be done with caution, however, as psychiatric diagnoses and psychosocial stressors may result in poorer outcomes of lifestyle interventions. Future development of lifestyle programs targeting AIWG should be designed for and tested in relevant psychiatric populations. Mixed data supports the effectiveness of lifestyle interventions in adults receiving AP treatment. A recent meta-analysis, however, reported a significant reduction in body weight after exercise initiation with a large effect size (SMD = −0.96), concluding that lifestyle interventions remain the most effective method to improve physical health outcomes in patients with schizophrenia (268). Compared to adults, pediatric patients present some unique challenges and advantages. Youth may not cognitively appreciate risk, may be less self-motivated to comply with prevention measures, and may be resistant to lifestyle interventions. On the other hand, caregivers are in a position to exercise considerable influence over diet, nutrition, and lifestyle factors.

As SGAs may directly affect gut microbiota populations (245, 249–252, 269, 270) probiotics, prebiotics, and fecal transplants have been proposed as potential therapies to reduce adverse AP effects. Prebiotics have been shown to promote the growth of beneficial microbiota in humans resulting in suppressed appetite in youth (*n* = 42, age range = 7–12) (271), and probiotics are associated with improved gastrointestinal function in patients with schizophrenia (272, 273). While a promising and novel strategy, further research efforts are necessary to explore specific gut microflora that could alleviate AP side effects. Animal models have aided in this effort. Probiotics were able to reverse weight gain and metabolic dysfunction resulting from olanzapine treatment in mice (247). The prebiotic B-GOS prevented weight gain in rats (274), and a prebiotic mixture reduced weight gain and decreased the putative obesogenic F:B ratio in mice (246). Fecal transplants from mice treated with risperidone have also been shown to reduce basal metabolic rate and increase weight gain in control mice (245). Nevertheless, to develop effective and safe interventions, preclinical studies and clinical trials in human subjects will be crucial. An exploratory study comparing children (*n* = 30, age range = 4–17) with extreme risperidone-induced weight gain vs. those without AIWG uncovered bile acid changes resulting from AP treatment and distinct bile acid profiles in subjects with vs. without weight gain (275, 276). Preliminary evidence suggests a potential link between bile acid changes and the gut microbiota. Interestingly, a similar mechanism was suggested by studies of metformin effects in diabetes (277).

### Pharmacological Treatments

Metformin, the most commonly used adjunctive medication targeting AIWG, is supported by the strongest evidence, but several other strategies have shown promise including glucagon-like peptide 1 receptor and histamine 1 receptor agonists. Unfortunately, most studies have been small and follow-up periods rarely exceed 6 months. Medications used to treat obesity in the general population have also been tried, and represent reasonable options, but these are often limited by intolerability of unpleasant side effects in psychiatric populations (278). In youth with metabolic abnormalities, insulin resistance, hyperglycemia and dyslipidemia, these medical sequelae are typically be managed by a pediatrician or endocrinologist. Studies have shown benefits of standard treatments, such as metformin and statins, in adults with antipsychotic related metabolic syndrome (279).

#### Metformin

Metformin, an anti-diabetic, has been studied extensively as a potential alleviator of AIWG and metabolic effects. Metformin has been shown to reduce metabolic effects in patients with schizophrenia spectrum disorders by decreasing hepatic gluconeogenesis, insulin resistance (i.e., improving insulin sensitivity) and total cholesterol (264, 268). Regulation of leptin sensitivity and hypothalamic signaling are also affected by metformin (280). Therefore, metformin may play a role in reducing caloric intake and fat storage (280). A 2014 meta-analysis of 40 studies on pharmacological interventions to combat adverse AP effects concluded that metformin should be the first choice for pharmacological treatment if non-pharmacological interventions have failed and switching to an AP with reduced potential for AIWG is not feasible (281). This meta-analysis was not focused on pediatric populations, but there have been several studies that have examined metformin adjunctive treatment in youth. In the Improving Parameters in Antipsychotic Child Treatment (IMPACT) trial of AIWG interventions, overweight youth (*n* = 127, age range = 8–19, mean age = 13.7) with a primary diagnosis of bipolar spectrum disorder, schizophrenia spectrum disorder, or psychotic depression were randomized to metformin treatment; AP switch to aripiprazole, perphenazine, or molindone; or continued current AP treatment (264). Both the metformin (moderate to large effect size 0.68) and AP switch (large effect size 0.81) group had significant reductions in BMI z-scores compared to the continued treatment control. More gastrointestinal complaints, however, were reported in the metformin group than the AP switch and control groups. Several additional studies support the benefit of metformin for pediatric SGA treatment. A randomized controlled trial (*n* = 61, age range = 6–17, mean age = 12.8) reported that metformin attenuated weight gain but did not affect metabolic measures (282), though given that no metabolic abnormalities were observed in either group, this is not surprising. Two small open label metformin trials reported weight loss in 5 out of 11 (age range = 10–18, mean age = 14) (283) and 15 out of 19 (age range = 10–18, mean age = 14.1) (284) patients. An open-label extension of one of the trials (282) found that this effect on anthropometric measures but not metabolic measures persists over long-term treatment, although they did note a non-significant decrease in hemoglobin A1c in both trial phases (285). One Iranian study failed to demonstrate a significant impact of metformin on AIWG prevention over a 12-week treatment period but did note significant effects over the first 4-weeks of treatment and a positive trend at 12 weeks (*n* = 49, mean age = 10.1) (286). Authors acknowledge that lower doses of metformin were used compared to other studies, which may explain conflicting results. Metformin may be more effective in first-episode psychosis patients in comparison to those receiving chronic treatment (287). Some have proposed that timing is critical and early use of metformin may improve outcomes through prevention rather than correction of weight gain (288). Lessons learned from metformin's modest efficacy may spur the testing and development of new approaches in the future.

### Glucagon-Like Peptide 1 Receptor Agonists (GLP1RA)

GLP1RAs, another medication class borrowed from diabetes treatment and associated with weight loss, have been examined as potential adjuncts to reduce AIWG and metabolic dysfunction (289). This approach is supported by evidence that serum GLP1 increases with SGA treatment (290) and is associated with both hyperglycemia as well as insulin resistance (291). A meta-analysis of 3 trials of adults receiving adjunctive GLP1RA along with an AP demonstrated reduced HbA1c, fasting blood glucose and BMI (292). This meta-analysis only consisted of 164 patients, underscoring the need for larger trials. Pediatric trials of efficacy in AIWG are lacking but warranted given promising effects of GL1RA treatment in adolescents with severe obesity (293). Until recently, GLP1RAs have been limited by a subcutaneous formulation, but the 2019 approval of an oral agent, semaglutide, could drive an expanded role for this medication class in the future.

#### Betahistine

Betahistine, an agonist of the H1 histamine receptor has been shown to reduce or attenuate olanzapine-induced AIWG in adults (121, 294, 295), as discussed previously. Many APs are antagonists at the H1 receptor, especially those with high propensity to cause AIWG. To our knowledge, only one study (296) has included pediatric patients (*n* = 12 of 51 total patients, age range 12-17). In this sample, betahistine tempered weight gain in participants receiving the strongly antihistaminergic APs olanzapine and clozapine, but not for those taking other APs with lower H1 potency. The study found no moderating effect of age (i.e., adolescent vs. adult) but did not analyze the adolescent population separately. Mechanistic queries of protective effects against AIWG conferred by potent H1 antagonists have been explored in several animal models (294, 297–302). The exact mechanism underlying beneficial effects is unclear, however, since adjunctive treatment with betahistine attenuates H1-NPY, H1-AMPK, and H1-POMC signaling and increases H3-mediated release of histamine. Further, in a rat model, betahistine was shown to reverse the upregulated dopamine D2R expression that typically results from olanzapine treatment, while not interfering with AP effects at serotonergic receptors in brain regions associated with AP efficacy (302). Thus, betahistine may reduce the increased D2 sensitivity associated with AP treatment but its potential interference with AP efficacy requires thorough investigation.

Other drugs that have been tested to reduce AIWG, largely in adults, include reboxantine, topiramate, and amantadine. When combined with betahistine, reboxantine, a norepinephrine reuptake inhibitor was shown to be effective in attenuating olanzapine-induced weight gain (294) and appetite (24) in adults. A meta-analysis of 10 studies of adjunctive topiramate (an antiepileptic drug known to reduce appetite) for AIWG, found topiramate mitigated weight gain in AP-treated adults (303). In a medical record review, there was an overall reduction in BMI for 47 child and adolescent psychiatric patients (mean age = 13.4) receiving topiramate and another anticonvulsant, zonisamide (304). Future efforts are needed to investigate these anticonvulsant adjunctive treatments to combat AIWG in pediatric patients.

The “natural,” over-the-counter supplement with the best evidence to mitigate AIWG and metabolic effects is melatonin. As discussed previously, melatonin plays a key role in energy homeostasis as well as central and peripheral insulin action (305). Reduction in melatonin production has been associated with insulin resistance, glucose intolerance, and metabolic disease (305). A recent meta-analysis of both adult and adolescent studies supported clinical use of melatonin and melatonin receptor agonists as adjuncts to mitigate AIWG and metabolic effects (175). In adolescents (*n* = 38) diagnosed with bipolar disorder receiving olanzapine and lithium combination therapy, melatonin as compared to placebo attenuated increases in cholesterol level and systolic blood pressure (306). In a smaller cohort (*n* = 19, mean age ~14), although not reaching significance, melatonin as compared to placebo reduced weight gain in the context of olanzapine and lithium combination therapy (307). Because of its role in the regulation of circadian rhythms (308), it is possible that positive metabolic effects may be secondary to beneficial effects on sleep, which has been show to play a role in obesity (309). Interestingly, co-administration of melatonin appears more effective with risperidone and quetiapine, agents with intermediate risk of AIWG, compared to olanzapine and clozapine, agents with the highest risk (175). If confirmed in larger studies, melatonin represents a relatively benign pharmacological intervention for youth.

## Clinical Guidelines for the Assessment, Prevention, and Treatment of Metabolic Adverse Effects of Antipsychotic Medications in Youth

**Figure 3:**
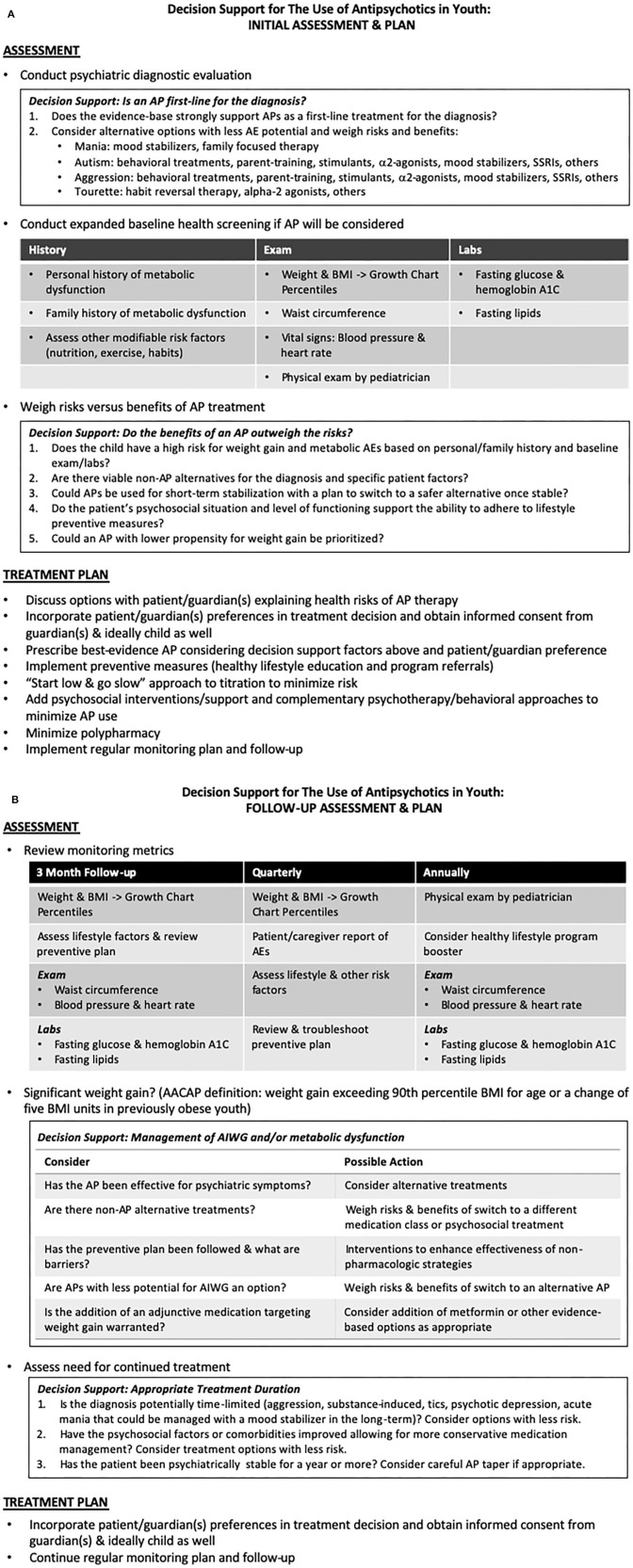
**(A,B)** Clinical guideline and decision support for the assessment, prevention, and treatment of metabolic adverse effects of antipsychotic medications in youth. Adapted from the American Academy of Child and Adolescent Psychiatry (7) and American Psychiatric Association and American Diabetes Association Guidelines (70). **(A)** Initial Assessment and Plan. **(B)** Follow-up Assessment and Plan.

Clinical practice guidelines are published by various groups, organizations, and experts. We present an overview of those guidelines most relevant to pediatric psychiatry, the AACAP Practice Parameters for Schizophrenia (7) and Aggression (14), supplemented with additional recommendations from the American Psychiatric Association (70, 82) and other adult sources (86, 278). The AACAP guidelines emphasize a comprehensive baseline assessment and treatment plan (Figure 3A) followed by responsible regular monitoring and follow-up (Figure 3B).

If initial psychiatric evaluation prompts the consideration of AP therapy, further baseline assessment should include expanded history (personal and family history of diabetes, hyperlipidemia, or previous response or adverse events associated with APs), physical exam (vital signs including blood pressure and heart rate, weight and BMI with determination of pediatric growth chart percentiles, and waist circumferences) and baseline laboratory measurements (fasting lipids, glucose and hemoglobin A1C).

The risks, benefits and alternatives of an AP should be assessed, weighed, and discussed (and informed consent obtained) with the guardian(s) and child/adolescent if possible. Even if an AP is a first-line treatment for the patient's diagnosis, behavioral treatments, psychosocial interventions, and medication alternatives should be considered. Behavioral and psychosocial approaches can improve outcomes and potentially reduce AP burden. Expected duration of therapy may also influence treatment planning, as it may be possible to minimize long-term AP use by switching to a safer medication or non-pharmacological treatment once an acute crisis has passed. If it is determined that benefits of an antipsychotic outweigh risks, the use of an AP with lower AIWG potential may be appropriate depending on diagnostic and patient-specific factors.

Once an AP is selected, a standard “start low and go slow” approach should be implemented. The administration of multiple concomitant psychotropic medications should be minimized, especially avoiding the concurrent use of multiple APs. Education and counseling on healthy lifestyle choices as preventive measures against weight gain and metabolic effects should be provided. Formal referral to a dietician or healthy lifestyle program may be warranted in higher risk or already overweight patients. An AACAP Facts for Families Sheet on “Weight Gain from Medication: Prevention and Management” is available on the AACAP website at https://www.aacap.org/AACAP/Families_and_Youth/Facts_for_Families/FFF-Guide/Preventing-and-Managing-Medication-Related-Weight-094.aspx.

Given the high risk of AIWG in youth taking APs, frequent monitoring of AEs must occur. Monitoring for AIWG in psychiatric patients using self-reported awareness is less effective than objective measurement (310). AACAP advises following the joint consensus recommendations of the American Diabetes Association and the American Psychiatric Association (Figure 3B) to monitor BMI quarterly and blood pressure, fasting blood glucose and fasting lipid profiles at 3 months and then annually thereafter (70). Since BMI distribution varies over typical development, BMI should be normed with respect to age and translated to percentiles. Developmentally normed growth charts can be found at the Center for Disease Control website (www.cdc.gov/growthcharts), and/or percentile calculators can be found online or in mobile app form. Despite strong guidelines, a 2016 review estimates that 70% of patients taking APs in the US fail to be screened or treated for metabolic AEs (311). The continued need for AP treatment should be regularly evaluated, as the appropriateness of long-term use will vary based on the severity of symptoms, psychosocial environment, availability of safer evidence-based options, and the natural course of the illness being treated.

Consideration of weight management interventions and increased regularity of blood glucose and lipid levels should be implemented if AIWG exceeds 90th percentile BMI for age, or a change occurs of 5 BMI units in patients already obese at baseline. Other contributors to weight gain and metabolic syndrome should be explored (312). A review and troubleshooting of lifestyle interventions may be adequate to curb weight gain, but in cases where lifestyle modification is insufficient, 2 main strategies exist for pharmacological intervention. First, a switch from an AP with higher to lower weight gain potential may be appropriate with careful attention to the risk of psychiatric relapse. The effectiveness of the current AP is an important factor. As reviewed previously, an AP switch strategy is supported by studies in both youth (264) and adults (313, 314); however, methodological problems, including high incidence of drug discontinuation and study attrition compounded by the use of per protocol data analysis, confound many of these trials and limit their application. Prior to switching, patients/families should be informed of the potential risk of relapse. Gradual cross-taper over several weeks is recommended to minimize this risk. If a switch to a lower risk AP is not appropriate or preferred, addition of an evidence-based adjunctive medication is a reasonable option with relatively low risk. As discussed above, metformin is currently the agent with the best support; however, several promising leads and novel alternatives are being developed and tested.

## Conclusions and Future Directions

SGA prescription has become the standard of care for children and adolescents with psychotic disorders as well as a frequent therapeutic employed, both with FDA-approval and off-label, for a range of psychiatric disorders. These APs have proven to be effective to reduce psychiatric symptoms but result in AEs, chiefly AIWG and metabolic effects. Despite robust research efforts to reveal underlying mechanisms, it is unclear how to maintain AP efficacy while reducing serious side effects.

The pharmacological interventions that have been proposed and investigated to date are limited. Interventions such as anti-diabetic and anti-convulsant medications are not biologically targeted treatments but rather repurposed based on incidental observations of weight loss or metabolic improvement when these medications are used for other conditions. Thus, these serendipitous positive effects may simply balance metabolic dysfunction rather than directly correct the underlying lesion driving these AEs. Additionally, drugs targeting obesity in the general population may be relevant to AIWG. Promising drugs that warrant further testing in AIWG include 5-HT_2C_R agonist lorcaserin (315), fat absorption blocker orlistat (316), and melanin concentrating hormone receptor 1 antagonists (317), as well as combination treatment of naltrexone and bupropion to curb craving (318). While obesity drugs are a Big Pharma priority, lack of mechanistic clarity underlying obesity and frequent prohibitive AEs have stalled progress. Future studies should take care to use high-quality study designs, including randomized controlled trials with intent-to-treat analysis, and provide effect sizes in addition to significance measures to convey the clinical utility of potential treatments.

Efforts to reduce AEs for pediatric patients undergoing SGA treatment will require mechanistic studies that illuminate a clearer, definitive conception of their biological underpinnings. Progress in neuroendocrine, genetic, and microbiome related mechanisms of AIWG lay a foundation for developing interventions to combat unwanted AEs. Manipulation of energy balance pathways in animal models can reveal potential avenues for human translation. Large-scale genomic and microbiome studies in both adult and pediatric patients can also yield links to biology. Further understanding of a healthy gut microbiome and effective manipulation strategies may expand psychotherapeutic modalities. Epigenetic changes produced by AP exposure, exploration of which remains rudimentary, may also contribute to compounding of genetic and environmental risk. A mechanistic appreciation of metabolic AEs will not only inform interventions to reduce or prevent side effects, but ultimately drive the design of specific therapies that can target psychiatric symptoms without inflicting harm.

The ever-expanding development of new technologies has the potential to considerably advance both discovery and intervention. Computational analysis of electronic medical records and machine learning approaches will generate and test new data-driven hypotheses. Similarly, wearable devices can collect objective data, such as patterns of activity, speech, sleep and biological metrics, that will facilitate clinician monitoring and feed big data approaches. Wearable devices and mobile apps can also be used to enhance patient engagement and motivation with lifestyle interventions and improve treatment adherence.

These diverse approaches can eventually explain the large, individual variability in risk for AIWG and metabolic effects and fuel precision medicine algorithms. The precision psychiatry model of the future seeks to incorporate demographic, genetic, epigenetic, biomarker, psychosocial, and other information to achieve a molecular diagnosis and a personalized risk assessment. This approach can match the individual patient with a data-driven treatment plan, thus boosting adherence, preventing AEs, and optimizing patient outcomes.

## Author Contributions

ML performed the primary literature search and prepared the first and final draft of the manuscript, figures, and table. EN defined the scope of the topic, authored the abstract, and edited the manuscript, figures, and tables.

## Conflict of Interest

EN serves on the Medical Advisory Board for the Tourette Association of America and Teva Pharmaceuticals and the Scientific Advisory Board for Myriad Genetics. The remaining author declares that the research was conducted in the absence of any commercial or financial relationships that could be construed as a potential conflict of interest.

## References

[1] Solmi M Murru A Pacchiarotti I Undurraga J Veronese N Fornaro M. Safety, tolerability, and risks associated with first- and second-generation antipsychotics: a state-of-the-art clinical review. Ther Clin Risk Manag. (2017) 13:757–77. 10.2147/TCRM.S117321 28721057 PMC5499790

[2] Seida JC Schouten JR Boylan K Newton AS Mousavi SS Beaith A. Antipsychotics for children and young adults: a comparative effectiveness review. Pediatrics. (2012) 129:e771–784. 10.1542/peds.2011-2158 22351885

[3] Fusar-Poli P Papanastasiou E Stahl D Rocchetti M Carpenter W Shergill S. Treatments of negative symptoms in schizophrenia: meta-analysis of 168 randomized placebo-controlled trials. Schizophr Bull. (2015) 41:892–9. 10.1093/schbul/sbu170 25528757 PMC4466178

[4] Alexander GC Gallagher SA Mascola A Moloney RM Stafford RS. Increasing off-label use of antipsychotic medications in the United States, 1995-2008. Pharmacoepidemiol Drug Saf . (2011) 20:177–84. 10.1002/pds.2082 21254289 PMC3069498

[5] Patel NC Crismon ML Hoagwood K Johnsrud MT Rascati KL Wilson JP. Trends in the use of typical and atypical antipsychotics in children and adolescents. J Am Acad Child Adolesc Psychiatry. (2005) 44:548–56. 10.1097/01.chi.0000157543.74509.c8 15908837

[6] Kumar S Kelly AS. Review of childhood obesity: from epidemiology, etiology, and comorbidities to clinical assessment and treatment. Mayo Clin Proc. (2017) 92:251–65. 10.1016/j.mayocp.2016.09.017 28065514

[7] McClellan J Stock S American Academy C and Adolescent Psychiatry Committee on Quality I. Practice parameter for the assessment and treatment of children and adolescents with schizophrenia. J Am Acad Child Adolesc Psychiatry. (2013) 52:976–90. 10.1016/j.jaac.2013.02.008 23972700

[8] Steinhausen HC. Recent international trends in psychotropic medication prescriptions for children and adolescents. Eur Child Adolesc Psychiatry. (2015) 24:635–40. 10.1007/s00787-014-0631-y 25378107

[9] Piovani D Clavenna A Bonati M. Prescription prevalence of psychotropic drugs in children and adolescents: an analysis of international data. Eur J Clin Pharmacol. (2019) 75:1333–46. 10.1007/s00228-019-02711-3 31270564

[10] Halfdanarson O Zoega H Aagaard L Bernardo M Brandt L Fuste AC. International trends in antipsychotic use: a study in 16 countries, 2005-2014. Eur Neuropsychopharmacol. (2017) 27:1064–76. 10.1016/j.euroneuro.2017.07.001 28755801

[11] Olfson M Blanco C Liu SM Wang S Correll CU. National trends in the office-based treatment of children, adolescents, and adults with antipsychotics. Arch Gen Psychiatry. (2012) 69:1247–56. 10.1001/archgenpsychiatry.2012.647 22868273

[12] Stigler KA Potenza MN McDougle CJ. Tolerability profile of atypical antipsychotics in children and adolescents. Paediatr Drugs. (2001) 3:927–42. 10.2165/00128072-200103120-00005 11772153

[13] Simeon J Milin R Walker S. A retrospective chart review of risperidone use in treatment-resistant children and adolescents with psychiatric disorders. Prog Neuropsychopharmacol Biol Psychiatry. (2002) 26:267–75. 10.1016/S0278-5846(01)00264-0 11817503

[14] Pappadopulos E Macintyre Ii JC Crismon ML Findling RL Malone RP Derivan A. Treatment recommendations for the use of antipsychotics for aggressive youth (TRAAY). Part II J Am Acad Child Adolesc Psychiatry. (2003) 42:145–61. 10.1097/00004583-200302000-00008 12544174

[15] Cooper WO Hickson GB Fuchs C Arbogast PG Ray WA. New users of antipsychotic medications among children enrolled in TennCare. Arch Pediatr Adolesc Med. (2004) 158:753–9. 10.1001/archpedi.158.8.753 15289247

[16] DelBello M Grcevich S. Phenomenology and epidemiology of childhood psychiatric disorders that may necessitate treatment with atypical antipsychotics. J Clin Psychiatry. (2004) 65 Suppl 6:12–9. 15104522

[17] Turgay A. Treatment of comorbidity in conduct disorder with attention-deficit hyperactivity disorder (ADHD). Essent Psychopharmacol. (2005) 6:277–90. Available online at: https://europepmc.org/article/med/16222912 16222912

[18] Glied S Cuellar AE. Trends and issues in child and adolescent mental health. Health Aff. (2003) 22:39–50. 10.1377/hlthaff.22.5.39 14515880

[19] Kalverdijk LJ Tobi H van den Berg PB Buiskool J Wagenaar L Minderaa RB. Use of antipsychotic drugs among dutch youths between 1997 and 2005. Psychiatr Serv. (2008) 59:554–60. 10.1176/ps.2008.59.5.554 18451016

[20] Rani F Murray ML Byrne PJ Wong IC. Epidemiologic features of antipsychotic prescribing to children and adolescents in primary care in the United Kingdom. Pediatrics. (2008) 121:1002–9. 10.1542/peds.2007-2008 18450906

[21] Pringsheim T Panagiotopoulos C Davidson J Ho J Group CG. Evidence-based recommendations for monitoring safety of second generation antipsychotics in children and youth. J Can Accad Child Adolesc Psychiatry. (2011) 20:218–233. 10.1093/pch/16.9.581 21804853 PMC3143700

[22] Zito JM Burcu M Ibe A Safer DJ Magder LS. Antipsychotic use by medicaid-insured youths: impact of eligibility and psychiatric diagnosis across a decade. Psychiatr Serv. (2013) 64:223–9. 10.1176/appi.ps.201200081 23242390

[23] Bachmann CJ Lempp T Glaeske G Hoffmann F. Antipsychotic prescription in children and adolescents: an analysis of data from a German statutory health insurance company from 2005 to 2012. Dtsch Arztebl Int. (2014) 111:25–34. 10.3238/arztebl.2014.0025 24606780 PMC3950759

[24] Dayabandara M Hanwella R Ratnatunga S Seneviratne S Suraweera C de Silva VA. Antipsychotic-associated weight gain: management strategies and impact on treatment adherence. Neuropsychiatr Dis Treat. (2017) 13:2231–41. 10.2147/NDT.S113099 28883731 PMC5574691

[25] Kjosavik SR Gillam MH Roughead EE. Average duration of treatment with antipsychotics among concession card holders in Australia. Aust N Z J Psychiatry. (2017) 51:719–26. 10.1177/0004867417691851 28195003

[26] Pringsheim T Lam D Patten SB. The pharmacoepidemiology of antipsychotic medications for Canadian children and adolescents: 2005-2009. J Child Adolesc Psychopharmacol. (2011) 21:537–43. 10.1089/cap.2010.0145 22136092

[27] Pennap D Zito JM Santosh PJ Tom SE Onukwugha E Magder LS. Patterns of early mental health diagnosis and medication treatment in a medicaid-insured birth cohort. JAMA Pediatr. (2018) 172:576–84. 10.1001/jamapediatrics.2018.0240 29710205 PMC6137539

[28] Findling RL Johnson JL McClellan J Frazier JA Vitiello B Hamer RM. Double-blind maintenance safety and effectiveness findings from the treatment of early-onset schizophrenia spectrum (TEOSS) study. J Am Acad Child Adolesc Psychiatry. (2010) 49:583–94; quiz 632. 10.1016/j.jaac.2010.03.013 20494268 PMC2882800

[29] Noguera A Ballesta P Baeza I Arango C de la Serna E Gonzalez-Pinto A. Twenty-four months of antipsychotic treatment in children and adolescents with first psychotic episode: discontinuation and tolerability. J Clin Psychopharmacol. (2013) 33:463–71. 10.1097/JCP.0b013e3182962480 23771198

[30] Upadhyay N Patel A Chan W Aparasu RR Ochoa-Perez M Sherer JT. Reversibility of psychotropic medication induced weight gain among children and adolescents with bipolar disorders. Psychiatry Res. (2019) 276:151–9. 10.1016/j.psychres.2019.05.005 31085419

[31] Lindsay RL Leone S Aman MG. Discontinuation of risperidone and reversibility of weight gain in children with disruptive behavior disorders. Clin Pediatr. (2004) 43:437–44. 10.1177/000992280404300504 15208748

[32] Reyes M Buitelaar J Toren P Augustyns I Eerdekens M. A randomized, double-blind, placebo-controlled study of risperidone maintenance treatment in children and adolescents with disruptive behavior disorders. Am J Psychiatry. (2006) 163:402–10. 10.1176/appi.ajp.163.3.402 16513860

[33] Calarge CA Nicol G Schlechte JA Burns TL. Cardiometabolic outcomes in children and adolescents following discontinuation of long-term risperidone treatment. J Child Adolesc Psychopharmacol. (2014) 24:120–9. 10.1089/cap.2013.0126 24725198 PMC3993060

[34] Pillay J Boylan K Newton A Hartling L Vandermeer B Nuspl M. Harms of antipsychotics in children and young adults: a systematic review update. Can J Psychiatry. (2018) 63:661–78. 10.1177/0706743718779950 29865900 PMC6187435

[35] Maayan L Correll CU. Weight gain and metabolic risks associated with antipsychotic medications in children and adolescents. J Child Adolesc Psychopharmacol. (2011) 21:517–35. 10.1089/cap.2011.0015 22166172

[36] Grajales D Ferreira V Valverde AM. Second-generation antipsychotics and dysregulation of glucose metabolism: beyond weight gain. Cells. (2019) 8:1336. 10.3390/cells8111336 31671770 PMC6912706

[37] Menard ML Thummler S Giannitelli M Cruzel C Bonnot O Cohen D. Incidence of adverse events in antipsychotic-naive children and adolescents treated with antipsychotic drugs: Results of a multicenter naturalistic study (ETAPE). Eur Neuropsychopharmacol. (2019) 29:1397–407. 10.1016/j.euroneuro.2019.10.006 31699516

[38] Weihe P Weihrauch-Bluher S. Metabolic syndrome in children and adolescents: diagnostic criteria, therapeutic options and perspectives. Curr Obes Rep. (2019) 8:472–9. 10.1007/s13679-019-00357-x 31691175

[39] Koskinen J Magnussen CG Sinaiko A Woo J Urbina E Jacobs DR Jr. Childhood Age and associations between childhood metabolic syndrome and adult risk for metabolic syndrome, type 2 diabetes mellitus and carotid intima media thickness: the international childhood cardiovascular cohort consortium. J Am Heart Assis. (2017) 6. 10.1161/JAHA.117.005632 28862940 PMC5586423

[40] Correll CU Manu P Olshanskiy V Napolitano B Kane JM Malhotra AK. Cardiometabolic risk of second-generation antipsychotic medications during first-time use in children and adolescents. JAMA. (2009) 302:1765–73. 10.1001/jama.2009.1549 19861668 PMC3055794

[41] Kryzhanovskaya LA Xu W Millen BA Acharya N Jen KY Osuntokun O. Comparison of long-term (at least 24 weeks) weight gain and metabolic changes between adolescents and adults treated with olanzapine. J Child Adolesc Psychopharmacol. (2012) 22:157–65. 10.1089/cap.2010.0020 22372514

[42] Hammerman A Dreiher J Klang SH Munitz H Cohen AD Goldfracht M. Antipsychotics and diabetes: an age-related association. Ann Pharmacother. (2008) 42:1316–22. 10.1345/aph.1L015 18664607

[43] Liao CH Chang CS Wei WC Chang SN Liao CC Lane HY. Schizophrenia patients at higher risk of diabetes, hypertension and hyperlipidemia: a population-based study. Schizophr Res. (2011) 126:110–6. 10.1016/j.schres.2010.12.007 21216567

[44] Nielsen RE Laursen MF Vernal DL Bisgaard C Jakobsen H Steinhausen HC. Risk of diabetes in children and adolescents exposed to antipsychotics: a nationwide 12-year case-control study. J Am Acad Child Adolesc Psychiatry. (2014) 53:971–9.e976. 10.1016/j.jaac.2014.04.023 25151420

[45] Rubin DM Kreider AR Matone M Huang YS Feudtner C Ross ME. Risk for incident diabetes mellitus following initiation of second-generation antipsychotics among Medicaid-enrolled youths. JAMA Pediatr. (2015) 169:e150285. 10.1001/jamapediatrics.2015.0285 25844991

[46] McIntyre RS Jerrell JM. Metabolic and cardiovascular adverse events associated with antipsychotic treatment in children and adolescents. Arch Pediatr Adolesc Med. (2008) 162:929–35. 10.1001/archpedi.162.10.929 18838645

[47] Jerrell JM Tripathi A Rizvi AA McIntyre RS. The risk of developing type 2 diabetes mellitus associated with psychotropic drug use in children and adolescents: a retrospective cohort analysis. Prim Care Companion CNS Disord. (2012) 14:11m01185. 10.4088/PCC.11m01185 22690363 PMC3357575

[48] Ostbye T Curtis LH Masselink LE Hutchison S Wright A Dans PE. Atypical antipsychotic drugs and diabetes mellitus in a large outpatient population: a retrospective cohort study. Pharmacoepidemiol Drug Saf . (2005) 14:407–15. 10.1002/pds.1016 15372671

[49] Andrade SE Lo JC Roblin D Fouayzi H Connor DF Penfold RB. Antipsychotic medication use among children and risk of diabetes mellitus. Pediatrics. (2011) 128:1135–41. 10.1542/peds.2011-0855 22106077

[50] Jasik CB Lustig RH. Adolescent obesity and puberty: the “perfect storm”. Ann N Y Acad Sic. (2008) 1135:265–79. 10.1196/annals.1429.009 18574233

[51] Ripoli C Pinna AP Podda F Zanni R Tronci MG Nurchi AM. Second-generation antipsychotic and diabetes mellitus in children and adolescents. Pediatr Med Chir. (2017) 39:149. 10.4081/pmc.2017.149 29502389

[52] Koller EA Doraiswamy PM. Olanzapine-associated diabetes mellitus. Pharmacotherapy. (2002) 22:841–52. 10.1592/phco.22.11.841.33629 12126218

[53] Sikich L Frazier JA McClellan J Findling RL Vitiello B Ritz L. Double-blind comparison of first- and second-generation antipsychotics in early-onset schizophrenia and schizo-affective disorder: findings from the treatment of early-onset schizophrenia spectrum disorders (TEOSS) study. Am J Psychiatry. (2008) 165:1420–31. 10.1176/appi.ajp.2008.08050756 18794207 PMC12860492

[54] Ucok A Gaebel W. Side effects of atypical antipsychotics: a brief overview. World Psychiatry. (2008) 7:58–62. 10.1002/j.2051-5545.2008.tb00154.x 18458771 PMC2327229

[55] Calarge CA Acion L Kuperman S Tansey M Schlechte JA. Weight gain and metabolic abnormalities during extended risperidone treatment in children and adolescents. J Child Adolesc Psychopharmacol. (2009) 19:101–9. 10.1089/cap.2008.007 19364288 PMC2715008

[56] Calarge CA Xie D Fiedorowicz JG Burns TL Haynes WG. Rate of weight gain and cardiometabolic abnormalities in children and adolescents. J Pediatr. (2012) 161:1010–5. 10.1016/j.jpeds.2012.05.051 22738944 PMC3461238

[57] Musil R Obermeier M Russ P Hamerle M. Weight gain and antipsychotics: a drug safety review. Expert Opin Drug Saf . (2015) 14:73–96. 10.1517/14740338.2015.974549 25400109

[58] Eapen V John G. Weight gain and metabolic syndrome among young patients on antipsychotic medication: what do we know and where do we go? Australas Psychiatry. (2011) 19:232–5. 10.3109/10398562.2010.539609 21682621

[59] McCracken JT McGough J Shah B Cronin P Hong D Aman MG. Risperidone in children with autism and serious behavioral problems. N Engl J Med. (2002) 347:314–21. 10.1056/NEJMoa013171 12151468

[60] Bobo WV Cooper WO Stein CM Olfson M Graham D Daugherty J. Antipsychotics and the risk of type 2 diabetes mellitus in children and youth. JAMA Psychiatry. (2013) 70:1067–75. 10.1001/jamapsychiatry.2013.2053 23965896

[61] Burcu M Zito JM Safer DJ Magder LS dosReis S Shaya FT. Concomitant use of atypical antipsychotics with other psychotropic medication classes and the risk of type 2 diabetes mellitus. J Am Acad Child Adolesc Psychiatry. (2017) 56:642–51. 10.1016/j.jaac.2017.04.004 28735693

[62] Martinez-Ortega JM Funes-Godoy S Diaz-Atienza F Gutierrez-Rojas L Perez-Costillas L Gurpegui M. Weight gain and increase of body mass index among children and adolescents treated with antipsychotics: a critical review. Eur Child Adolesc Psychiatry. (2013) 22:457–79. 10.1007/s00787-013-0399-5 23503976

[63] Ratzoni G Gothelf D Brand-Gothelf A Reidman J Kikinzon L Gal G. Weight gain associated with olanzapine and risperidone in adolescent patients: a comparative prospective study. J Am Acad Child Adolesc Psychiatry. (2002) 41:337–43. 10.1097/00004583-200203000-00014 11886029

[64] Safer DJ. A comparison of risperidone-induced weight gain across the age span. J Clin Psychopharmacol. (2004) 24:429–36. 10.1097/01.jcp.0000130558.86125.5b 15232335

[65] Bretler T Weisberg H Koren O Neuman H. The effects of antipsychotic medications on microbiome and weight gain in children and adolescents. BMC Med. (2019) 17:112. 10.1186/s12916-019-1346-1 31215494 PMC6582584

[66] De Hert M Dobbelaere M Sheridan EM Cohen D Correll CU. Metabolic and endocrine adverse effects of second-generation antipsychotics in children and adolescents: a systematic review of randomized, placebo controlled trials and guidelines for clinical practice. Eur Psychiatry. (2011) 26:144–58. 10.1016/j.eurpsy.2010.09.011 21295450

[67] Alvarez-Jimenez M Gonzalez-Blanch C Crespo-Facorro B Hetrick S Rodriguez-Sanchez JM Perez-Iglesias R. Antipsychotic-induced weight gain in chronic and first-episode psychotic disorders: a systematic critical reappraisal. CNS Drugs. (2008) 22:547–62. 10.2165/00023210-200822070-00002 18547125

[68] Correll CU Robinson DG Schooler NR Brunette MF Mueser KT Rosenheck RA. Cardiometabolic risk in patients with first-episode schizophrenia spectrum disorders: baseline results from the RAISE-ETP study. JAMA Psychiatry. (2014) 71:1350–63. 10.1001/jamapsychiatry.2014.1314 25321337

[69] Jensen KG Correll CU Ruda D Klauber DG Decara MS Fagerlund B. Cardiometabolic adverse effects and its predictors in children and adolescents with first-episode psychosis during treatment with quetiapine-extended release versus aripiprazole: 12-week results from the tolerance and effect of antipsychotics in children and adolescents with psychosis (TEA) trial. J Am Acad Child Adolesc Psychiatry. (2019) 58:1062–78. 10.1016/j.jaac.2019.01.015 30858012

[70] American Diabetes A American Psychiatric A American Association of Clinical E North American Association for the Study O. Consensus development conference on antipsychotic drugs and obesity and diabetes. Diabetes Care. (2004) 27:596–601. 10.2337/diacare.27.2.596 14747245

[71] Fraguas D Merchan-Naranjo J Laita P Parellada M Moreno D Ruiz-Sancho A. Metabolic and hormonal side effects in children and adolescents treated with second-generation antipsychotics. J Clin Psychiatry. (2008) 69:1166–75. 10.4088/JCP.v69n0717 18588363

[72] Morrato EH Nicol GE Maahs D Druss BG Hartung DM Valuck RJ. Metabolic screening in children receiving antipsychotic drug treatment. Arch Pediatr Adolesc Med. (2010) 164:344–51. 10.1001/archpediatrics.2010.48 20368487

[73] Ho J Panagiotopoulos C McCrindle B Grisaru S Pringsheim T Group CG. Management recommendations for metabolic complications associated with second generation antipsychotic use in children and youth. J Can Accad Child Adolesc Psychiatry. (2011) 20:234–41. 21804854 PMC3143701

[74] Burcu M Zito JM Ibe A Safer DJ. Atypical antipsychotic use among Medicaid-insured children and adolescents: duration, safety, and monitoring implications. J Child Adolesc Psychopharmacol. (2014) 24:112–9. 10.1089/cap.2013.0094 24690011

[75] Pringsheim T Ho J Sarna JR Hammer T Patten S. Feasibility and relevance of antipsychotic safety monitoring in children with tourette syndrome: a prospective longitudinal study. J Clin Psychopharmacol. (2017) 37:498–504. 10.1097/JCP.0000000000000760 28816926

[76] Santosh PJ Bell L Fiori F Singh J. Pediatric Antipsychotic use and outcomes monitoring. J Child Adolesc Psychopharmacol. (2017) 27:546–54. 10.1089/cap.2015.0247 27607909

[77] Wakefield S Aligeti M Rachamallu V Baronia R Aynampudi R Parmar A. Metabolic monitoring of child and adolescent patients on atypical antipsychotics by psychiatrists and primary care providers. Am J Ther. (2020) 27:e425–e430. 10.1097/MJT.0000000000000853 30762589

[78] Krause M Zhu Y Huhn M Schneider-Thoma J Bighelli I Chaimani A. Efficacy, acceptability, and tolerability of antipsychotics in children and adolescents with schizophrenia: a network meta-analysis. Eur Neuropsychopharmacol. (2018) 28:659–74. 10.1016/j.euroneuro.2018.03.008 29802039

[79] Pozzi M Pisano S Marano G Carnovale C Bravaccio C Rafaniello C. Weight-change trajectories of pediatric outpatients treated with risperidone or aripiprazole in a naturalistic setting. J Child Adolesc Psychopharmacol. (2019) 29:133–40. 10.1089/cap.2018.0092 30452281

[80] Schoemakers RJ van Kesteren C van Rosmalen J Eussen M Dieleman HG Beex-Oosterhuis MM. No differences in weight gain between risperidone and aripiprazole in children and adolescents after 12 months. J Child Adolesc Psychopharmacol. (2019) 29:192–6. 10.1089/cap.2018.0111 30672720

[81] Simon V van Winkel R De Hert M. Are weight gain and metabolic side effects of atypical antipsychotics dose dependent? A literature review. J Clin Psychiatry. (2009) 70:1041–50. 10.4088/JCP.08r04392 19653979

[82] Keepers GA Fochtmann LJ Anzia JM Benjamin S Lyness JM Mojtabai R. The American psychiatric association practice guideline for the treatment of patients with schizophrenia. Am J Psychiatry. (2020) 177:868–72. 10.1176/appi.ajp.2020.177901 32867516

[83] van der Esch CCL Kloosterboer SM van der Ende J Reichart CG Kouijzer MEJ de Kroon MMJ. Risk factors and pattern of weight gain in youths using antipsychotic drugs. Eur Child Adolesc Psychiatry. (2020) 1–9. 10.1007/s00787-020-01614-4 32839872 PMC8310848

[84] Bowden CL. Atypical antipsychotic augmentation of mood stabilizer therapy in bipolar disorder. J Clin Psychiatry. (2005) 66 (Suppl. 3):12–9. 15762830

[85] Taylor JH Jakubovski E Gabriel D Bloch MH. Predictors and moderators of antipsychotic-related weight gain in the treatment of early-onset schizophrenia spectrum disorders study. J Child Adolesc Psychopharmacol. (2018) 28:474–84. 10.1089/cap.2017.0147 29920116 PMC6154761

[86] Cooper SJ Reynolds GP Barnes T England E Haddad PM Heald A. BAP guidelines on the management of weight gain, metabolic disturbances and cardiovascular risk associated with psychosis and antipsychotic drug treatment. J Psychopharmacol. (2016) 30:717–48. 10.1177/0269881116645254 27147592

[87] Nurmi EL Spilman SL Whelan F Scahill LL Aman MG McDougle CJ. Moderation of antipsychotic-induced weight gain by energy balance gene variants in the RUPP autism network risperidone studies. Transl Psychiatry. (2013) 3:e274. 10.1038/tp.2013.26 23799528 PMC3693401

[88] Maayan L Correll CU. Management of antipsychotic-related weight gain. Expert Rev Neurother. (2010) 10:1175–200. 10.1586/ern.10.85 20586697 PMC3501406

[89] Andersen SW Clemow DB Corya SA. Long-term weight gain in patients treated with open-label olanzapine in combination with fluoxetine for major depressive disorder. J Clin Psychiatry. (2005) 66:1468–76. 10.4088/JCP.v66n1118 16420086

[90] Gebhardt S Haberhausen M Heinzel-Gutenbrunner M Gebhardt N Remschmidt H Krieg JC. Antipsychotic-induced body weight gain: predictors and a systematic categorization of the long-term weight course. J Psychiatr Res. (2009) 43:620–6. 10.1016/j.jpsychires.2008.11.001 19110264

[91] Haack S Seeringer A Thurmann PA Becker T Kirchheiner J. Sex-specific differences in side effects of psychotropic drugs: genes or gender? Pharmacogenomics. (2009) 10:1511–26. 10.2217/pgs.09.102 19761372

[92] Pillinger T McCutcheon RA Vano L Mizuno Y Arumuham A Hindley G. Comparative effects of 18 antipsychotics on metabolic function in patients with schizophrenia, predictors of metabolic dysregulation, and association with psychopathology: a systematic review and network meta-analysis. Lancet Psychiatry. (2020) 7:64–77. 10.1016/S2215-0366(19)30416-X 31860457 PMC7029416

[93] Lee E Leung CM Wong E. Atypical antipsychotics and weight gain in Chinese patients: a comparison of olanzapine and risperidone. J Clin Psychiatry. (2004) 65:864–6. 10.4088/JCP.v65n0620 15291666

[94] Ader M Garvey WT Phillips LS Nemeroff CB Gharabawi G Mahmoud R. Ethnic heterogeneity in glucoregulatory function during treatment with atypical antipsychotics in patients with schizophrenia. J Psychiatr Res. (2008) 42:1076–85. 10.1016/j.jpsychires.2008.01.004 18295798 PMC3769976

[95] Zito JM Safer DJ Sai D Gardner JF Thomas D Coombes P. Psychotropic medication patterns among youth in foster care. Pediatrics. (2008) 121:e157–63. 10.1542/peds.2007-0212 18166534

[96] Curtis LH Masselink LE Ostbye T Hutchison S Dans PE Wright A. Prevalence of atypical antipsychotic drug use among commercially insured youths in the United States. Arch Pediatr Adolesc Med. (2005) 159:362–6. 10.1001/archpedi.159.4.362 15809391

[97] Crystal S Olfson M Huang C Pincus H Gerhard T. Broadened use of atypical antipsychotics: safety, effectiveness, and policy challenges. Health Aff. (2009) 28:w770–81. 10.1377/hlthaff.28.5.w770 19622537 PMC2896705

[98] Richelson E Souder T. Binding of antipsychotic drugs to human brain receptors focus on newer generation compounds. Life Sci. (2000) 68:29–39. 10.1016/S0024-3205(00)00911-5 11132243

[99] Kroeze WK Hufeisen SJ Popadak BA Renock SM Steinberg S Ernsberger P. H1-histamine receptor affinity predicts short-term weight gain for typical and atypical antipsychotic drugs. Neuropsychopharmacology. (2003) 28:519–26. 10.1038/sj.npp.1300027 12629531

[100] Ishibashi T Horisawa T Tokuda K Ishiyama T Ogasa M Tagashira R. Pharmacological profile of lurasidone, a novel antipsychotic agent with potent 5-hydroxytryptamine 7 (5-HT7) and 5-HT1A receptor activity. J Pharmacol Exp Ther. (2010) 334:171–81. 10.1124/jpet.110.167346 20404009

[101] Roerig JL Steffen KJ Mitchell JE. Atypical antipsychotic-induced weight gain: insights into mechanisms of action. CNS Drugs. (2011) 25:1035–59. 10.2165/11596300-000000000-00000 22133326

[102] Lett TA Wallace TJ Chowdhury NI Tiwari AK Kennedy JL Muller DJ. Pharmacogenetics of antipsychotic-induced weight gain: review and clinical implications. Mol Psychiatry. (2012) 17:242–66. 10.1038/mp.2011.109 21894153

[103] Nasrallah HA. Atypical antipsychotic-induced metabolic side effects: insights from receptor-binding profiles. Mol Psychiatry. (2008) 13:27–35. 10.1038/sj.mp.4002066 17848919

[104] Garcia-Serna R Ursu O Oprea TI Mestres J. iPHACE: integrative navigation in pharmacological space. Bioinformatics. (2010) 26:985–6. 10.1093/bioinformatics/btq061 20156991 PMC2844997

[105] Reynolds GP Kirk SL. Metabolic side effects of antipsychotic drug treatment–pharmacological mechanisms. Pharmacol Ther. (2010) 125:169–79. 10.1016/j.pharmthera.2009.10.010 19931306

[106] Besnard J Ruda GF Setola V Abecassis K Rodriguiz RM Huang XP. Automated design of ligands to polypharmacological profiles. Nature. (2012) 492:215–20. 10.1038/nature11691 23235874 PMC3653568

[107] Maeda K Sugino H Akazawa H Amada N Shimada J Futamura T. Brexpiprazole I: in vitro and in vivo characterization of a novel serotonin-dopamine activity modulator. J Pharmacol Exp Ther. (2014) 350:589–604. 10.1124/jpet.114.213793 24947465

[108] Fountoulakis KN Gazouli M Kelsoe J Akiskal H. The pharmacodynamic properties of lurasidone and their role in its antidepressant efficacy in bipolar disorder. Eur Neuropsychopharmacol. (2015) 25:335–42. 10.1016/j.euroneuro.2014.11.010 25596883

[109] O'Connor WT O'Shea SD. Clozapine and GABA transmission in schizophrenia disease models: establishing principles to guide treatments. Pharmacol Ther. (2015) 150:47–80. 10.1016/j.pharmthera.2015.01.005 25585121

[110] Siafis S Tzachanis D Samara M Papazisis G. Antipsychotic drugs: from receptor-binding profiles to metabolic side effects. Curr Neuropharmacol. (2018) 16:1210–23. 10.2174/1570159X15666170630163616 28676017 PMC6187748

[111] Correll CU. From receptor pharmacology to improved outcomes: individualising the selection, dosing, and switching of antipsychotics. Eur Psychiatry. (2010) 25 (Suppl. 2):S12–21. 10.1016/S0924-9338(10)71701-6 20620881

[112] Correll CU Lencz T Malhotra AK. Antipsychotic drugs and obesity. Trends Mol Med. (2011) 17:97–107. 10.1016/j.molmed.2010.10.010 21185230 PMC3053585

[113] Kapur S Zipursky R Jones C Remington G Houle S. Relationship between dopamine D(2) occupancy, clinical response, and side effects: a double-blind PET study of first-episode schizophrenia. Am J Psychiatry. (2000) 157:514–20. 10.1176/appi.ajp.157.4.514 10739409

[114] Huang XF Tan YY Huang X Wang Q. Effect of chronic treatment with clozapine and haloperidol on 5-HT(2A and 2C) receptor mRNA expression in the rat brain. Neurosci Res. (2007) 59:314–21. 10.1016/j.neures.2007.08.001 17868938

[115] Travis MJ Busatto GF Pilowsky LS Mulligan R Acton PD Gacinovic S. 5-HT2A receptor blockade in patients with schizophrenia treated with risperidone or clozapine. A SPET study using the novel 5-HT2A ligand 123I-5-I-R-91150. Br J Psychiatry. (1998) 173:236–41. 10.1192/bjp.173.3.236 9926100

[116] Leibowitz SF Alexander JT. Hypothalamic serotonin in control of eating behavior, meal size, and body weight. Biol Psychiatry. (1998) 44:851–64. 10.1016/S0006-3223(98)00186-3 9807640

[117] Herrick-Davis K Grinde E Teitler M. Inverse agonist activity of atypical antipsychotic drugs at human 5-hydroxytryptamine2C receptors. J Pharmacol Exp Ther. (2000) 295:226–32. 10991983

[118] Rauser L Savage JE Meltzer HY Roth BL. Inverse agonist actions of typical and atypical antipsychotic drugs at the human 5-hydroxytryptamine(2C) receptor. J Pharmacol Exp Ther. (2001) 299:83–9. 11561066

[119] Shapiro DA Renock S Arrington E Chiodo LA Liu LX Sibley DR. Aripiprazole, a novel atypical antipsychotic drug with a unique and robust pharmacology. Neuropsychopharmacology. (2003) 28:1400–11. 10.1038/sj.npp.1300203 12784105

[120] Lian J Huang XF Pai NB Deng C. Reduce the Olanzapine-Induced Body Weight Gain With Histamine H1 Receptor Agonist Betahistine in Rats. Oxford: Oxford University Press (2010).

[121] Barak N Beck Y Albeck JH. Betahistine decreases olanzapine-induced weight gain and somnolence in humans. J Psychopharmacol. (2016) 30:237–41. 10.1177/0269881115626349 26839321

[122] Kim SF Huang AS Snowman AM Teuscher C Snyder SH. From the Cover: Antipsychotic drug-induced weight gain mediated by histamine H1 receptor-linked activation of hypothalamic AMP-kinase. Proc Natl Acad Sci USA. (2007) 104:3456–9. 10.1073/pnas.0611417104 17360666 PMC1805549

[123] Ballon JS Pajvani U Freyberg Z Leibel RL Lieberman JA. Molecular pathophysiology of metabolic effects of antipsychotic medications. Trends Endocrinol Metab. (2014) 25:593–600. 10.1016/j.tem.2014.07.004 25190097

[124] Kim SH Nikolics L Abbasi F Lamendola C Link J Reaven GM. Relationship between body mass index and insulin resistance in patients treated with second generation antipsychotic agents. J Psychiatr Res. (2010) 44:493–8. 10.1016/j.jpsychires.2009.11.007 19962157 PMC2873096

[125] Li P Snyder GL Vanover KE. Dopamine Targeting Drugs for the Treatment of Schizophrenia: Past, Present and Future. Curr Top Med Chem. (2016) 16:3385–403. 10.2174/1568026616666160608084834 27291902 PMC5112764

[126] Kaur G Kulkarni SK. Studies on modulation of feeding behavior by atypical antipsychotics in female mice. Prog Neuropsychopharmacol Biol Psychiatry. (2002) 26:277–85. 10.1016/S0278-5846(01)00266-4 11817504

[127] Wang GJ Volkow ND Logan J Pappas NR Wong CT Zhu W. Brain dopamine and obesity. Lancet. (2001) 357:354–7. 10.1016/S0140-6736(00)03643-6 11210998

[128] Wang GJ Volkow ND Thanos PK Fowler JS. Similarity between obesity and drug addiction as assessed by neurofunctional imaging: a concept review. J Addict Dis. (2004) 23:39–53. 10.1300/J069v23n03_04 15256343

[129] Lane HY Liu YC Huang CL Chang YC Wu PL Lu CT. Risperidone-related weight gain: genetic and nongenetic predictors. J Clin Psychopharmacol. (2006) 26:128–34. 10.1097/01.jcp.0000203196.65710.2b 16633140

[130] Saiz PA Susce MT Clark DA Kerwin RW Molero P Arranz MJ. An investigation of the alpha1A-adrenergic receptor gene and antipsychotic-induced side-effects. Hum Psychopharmacol. (2008) 23:107–14. 10.1002/hup.903 17972277

[131] Hahn M Chintoh A Giacca A Xu L Lam L Mann S. Atypical antipsychotics and effects of muscarinic, serotonergic, dopaminergic and histaminergic receptor binding on insulin secretion in vivo: an animal model. Schizophr Res. (2011) 131:90–5. 10.1016/j.schres.2011.06.004 21696923

[132] Baeza I Vigo L de la Serna E Calvo-Escalona R Merchan-Naranjo J Rodriguez-Latorre P. The effects of antipsychotics on weight gain, weight-related hormones and homocysteine in children and adolescents: a 1-year follow-up study. Eur Child Adolesc Psychiatry. (2017) 26:35–46. 10.1007/s00787-016-0866-x 27209421

[133] Havel PJ. Peripheral signals conveying metabolic information to the brain: short-term and long-term regulation of food intake and energy homeostasis. Exp Biol Med. (2001) 226:963–77. 10.1177/153537020122601102 11743131

[134] Endomba FT Tankeu AT Nkeck JR Tochie JN. Leptin and psychiatric illnesses: does leptin play a role in antipsychotic-induced weight gain? Lipids Health Dis. (2020) 19:22. 10.1186/s12944-020-01203-z 32033608 PMC7006414

[135] Farr OM Gavrieli A Mantzoros CS. Leptin applications in 2015: what have we learned about leptin and obesity? Curr Opin Endocrinol Diabetes Obes. (2015) 22:353–9. 10.1097/MED.0000000000000184 26313897 PMC4610373

[136] Flak JN Myers MGJr. Minireview: CNS mechanisms of leptin action. Mol Endocrinol. (2016) 30:3–12. 10.1210/me.2015-1232 26484582 PMC4695630

[137] Gruzdeva O Borodkina D Uchasova E Dyleva Y Barbarash O. Leptin resistance: underlying mechanisms and diagnosis. Diabetes Metab Syndr Obes. (2019) 12:191–8. 10.2147/DMSO.S182406 30774404 PMC6354688

[138] Park HK Ahima RS. Physiology of leptin: energy homeostasis, neuroendocrine function and metabolism. Metab Clin Exp. (2015) 64:24–34. 10.1016/j.metabol.2014.08.004 25199978 PMC4267898

[139] Kahn BB Minokoshi Y. Leptin, GABA, and glucose control. Cell Metab. (2013) 18:304–6. 10.1016/j.cmet.2013.08.015 24011066 PMC4097301

[140] Stubbs B Wang AK Vancampfort D Miller BJ. Are leptin levels increased among people with schizophrenia versus controls? A systematic review and comparative meta-analysis. Psychoneuroendocrinology. (2016) 63:144–54. 10.1016/j.psyneuen.2015.09.026 26444588

[141] Monteleone P Fabrazzo M Tortorella A La Pia S Maj M. Pronounced early increase in circulating leptin predicts a lower weight gain during clozapine treatment. J Clin Psychopharmacol. (2002) 22:424–6. 10.1097/00004714-200208000-00015 12172344

[142] Reynolds GP Zhang ZJ Zhang XB. Association of antipsychotic drug-induced weight gain with a 5-HT2C receptor gene polymorphism. Lancet. (2002) 359:2086–7. 10.1016/S0140-6736(02)08913-4 12086765

[143] Gorobets LN. Contribution of leptin to the formation of neuroleptic obesity in patients with schizophrenia during antipsychotic therapy. Bull Exp Biol Med. (2008) 146:348–50. 10.1007/s10517-008-0294-0 19240857

[144] Lee AK Bishop JR. Pharmacogenetics of leptin in antipsychotic-associated weight gain and obesity-related complications. Pharmacogenomics. (2011) 12:999–1016. 10.2217/pgs.11.45 21787190 PMC3792568

[145] Brandl EJ Frydrychowicz C Tiwari AK Lett TA Kitzrow W Buttner S. Association study of polymorphisms in leptin and leptin receptor genes with antipsychotic-induced body weight gain. Prog Neuropsychopharmacol Biol Psychiatry. (2012) 38:134–41. 10.1016/j.pnpbp.2012.03.001 22426215

[146] Shen J Ge W Zhang J Zhu HJ Fang Y. Leptin−2548g/a gene polymorphism in association with antipsychotic-induced weight gain: a meta-analysis study. Psychiatr Danub. (2014) 26:145–51. 24909251

[147] Haupt DW Luber A Maeda J Melson AK Schweiger JA Newcomer JW. Plasma leptin and adiposity during antipsychotic treatment of schizophrenia. Neuropsychopharmacology. (2005) 30:184–91. 10.1038/sj.npp.1300563 15367925

[148] Templeman LA Reynolds GP Arranz B San L. Polymorphisms of the 5-HT2C receptor and leptin genes are associated with antipsychotic drug-induced weight gain in Caucasian subjects with a first-episode psychosis. Pharmacogenet Genomics. (2005) 15:195–200. 10.1097/01213011-200504000-00002 15864111

[149] von Wilmsdorff M Bouvier ML Henning U Schmitt A Gaebel W. The impact of antipsychotic drugs on food intake and body weight and on leptin levels in blood and hypothalamic ob-r leptin receptor expression in wistar rats. Clinics. (2010) 65:885–94. 10.1590/S1807-59322010000900012 21049217 PMC2954740

[150] Panariello F Polsinelli G Borlido C Monda M De Luca V. The role of leptin in antipsychotic-induced weight gain: genetic and non-genetic factors. J Obes. (2012) 2012:572848. 10.1155/2012/572848 22523667 PMC3317122

[151] Potvin S Zhornitsky S Stip E. Antipsychotic-induced changes in blood levels of leptin in schizophrenia: a meta-analysis. Can J Psychiatry. (2015) 60 (3 Suppl. 2):S26–34. 25886677 PMC4418620

[152] Misiak B Bartoli F Stramecki F Samochowiec J Lis M Kasznia J. Appetite regulating hormones in first-episode psychosis: a systematic review and meta-analysis. Neurosci Biobehav Rev. (2019) 102:362–70. 10.1016/j.neubiorev.2019.05.018 31121198

[153] Yadav A Kataria MA Saini V Yadav A. Role of leptin and adiponectin in insulin resistance. Clin Chim Acta. (2013) 417:80–4. 10.1016/j.cca.2012.12.007 23266767

[154] Shimomura I Funahashi T Takahashi M Maeda K Kotani K Nakamura T. Enhanced expression of PAI-1 in visceral fat: possible contributor to vascular disease in obesity. Nat Med. (1996) 2:800–3. 10.1038/nm0796-800 8673927

[155] Fried SK Bunkin DA Greenberg AS. Omental and subcutaneous adipose tissues of obese subjects release interleukin-6: depot difference and regulation by glucocorticoid. J Clin Endocrinol Metab. (1998) 83:847–50. 10.1210/jc.83.3.847 9506738

[156] Teff KL Kim SF. Atypical antipsychotics and the neural regulation of food intake and peripheral metabolism. Physiol Behav. (2011) 104:590–8. 10.1016/j.physbeh.2011.05.033 21664918 PMC3139777

[157] Volpato AM Zugno AI Quevedo J. Recent evidence and potential mechanisms underlying weight gain and insulin resistance due to atypical antipsychotics. Braz J Psychiatry. (2013) 35:295–304. 10.1590/1516-4446-2012-1052 24142093

[158] Masaki T Yoshimatsu H Chiba S Watanabe T Sakata T. Targeted disruption of histamine H1-receptor attenuates regulatory effects of leptin on feeding, adiposity, and UCP family in mice. Diabetes. (2001) 50:385–91. 10.2337/diabetes.50.2.385 11272151

[159] Shen L Wang DQ Tso P Jandacek RJ Woods SC Liu M. Apolipoprotein E reduces food intake via PI3K/Akt signaling pathway in the hypothalamus. Physiol Behav. (2011) 105:124–8. 10.1016/j.physbeh.2011.04.018 21536059 PMC3160520

[160] Kullmann S Kleinridders A Small DM Fritsche A Haring HU Preissl H. Central nervous pathways of insulin action in the control of metabolism and food intake. Lancet Diabetes Endocrinol. (2020) 8:524–34. 10.1016/S2213-8587(20)30113-3 32445739

[161] Tschritter O Preissl H Hennige AM Stumvoll M Porubska K Frost R. The cerebrocortical response to hyperinsulinemia is reduced in overweight humans: a magnetoencephalographic study. Proc Natl Acad Sci USA. (2006) 103:12103–8. 10.1073/pnas.0604404103 16877540 PMC1567704

[162] Barsh GS Schwartz MW. Genetic approaches to studying energy balance: perception and integration. Nat Rev Genet. (2002) 3:589–600. 10.1038/nrg862 12154382

[163] Kullmann S Frank S Heni M Ketterer C Veit R Haring HU. Intranasal insulin modulates intrinsic reward and prefrontal circuitry of the human brain in lean women. Neuroendocrinology. (2013) 97:176–82. 10.1159/000341406 22922661

[164] Schilling TM Ferreira de Sa DS Westerhausen R Strelzyk F Larra MF Hallschmid M. Intranasal insulin increases regional cerebral blood flow in the insular cortex in men independently of cortisol manipulation. Hum Brain Mapp. (2014) 35:1944–56. 10.1002/hbm.22304 23907764 PMC6869468

[165] Schmid V Kullmann S Gfrorer W Hund V Hallschmid M Lipp HP. Safety of intranasal human insulin: a review. Diabetes Obes Metab. (2018) 20:1563–77. 10.1111/dom.13279 29508509

[166] Ghasemi R Dargahi L Haeri A Moosavi M Mohamed Z Ahmadiani A. Brain insulin dysregulation: implication for neurological and neuropsychiatric disorders. Mol Neurobiol. (2013) 47:1045–65. 10.1007/s12035-013-8404-z 23335160

[167] Heilbronn LK Smith SR Ravussin E. The insulin-sensitizing role of the fat derived hormone adiponectin. Curr Pharm Des. (2003) 9:1411–8. 10.2174/1381612033454766 12769732

[168] Weyer C Funahashi T Tanaka S Hotta K Matsuzawa Y Pratley RE. Hypoadiponectinemia in obesity and type 2 diabetes: close association with insulin resistance and hyperinsulinemia. J Clin Endocrinol Metab. (2001) 86:1930–5. 10.1210/jcem.86.5.7463 11344187

[169] Kadowaki T Yamauchi T. Adiponectin and adiponectin receptors. Endocr Rev. (2005) 26:439–51. 10.1210/er.2005-0005 15897298

[170] Bartoli F Lax A Crocamo C Clerici M Carra G. Plasma adiponectin levels in schizophrenia and role of second-generation antipsychotics: a meta-analysis. Psychoneuroendocrinology. (2015) 56:179–89. 10.1016/j.psyneuen.2015.03.012 25827962

[171] Sentissi O Epelbaum J Olie JP Poirier MF. Leptin and ghrelin levels in patients with schizophrenia during different antipsychotics treatment: a review. Schizophr Bull. (2008) 34:1189–99. 10.1093/schbul/sbm141 18165262 PMC2632509

[172] Jin H Meyer JM Mudaliar S Jeste DV. Impact of atypical antipsychotic therapy on leptin, ghrelin, and adiponectin. Schizophr Res. (2008) 100:70–85. 10.1016/j.schres.2007.11.026 18206351 PMC2699769

[173] Firth J Teasdale SB Jackson SE Vancampfort D Siskind D Sarris J. Do reductions in ghrelin contribute towards antipsychotic-induced weight gain? Schizophr Res. (2019) 210:301–2. 10.1016/j.schres.2018.12.043 30595440

[174] Goetz RL Miller BJ. Meta-analysis of ghrelin alterations in schizophrenia: effects of olanzapine. Schizophr Res. (2019) 206:21–6. 10.1016/j.schres.2018.11.036 30528312

[175] Wang HR Woo YS Bahk WM. The role of melatonin and melatonin agonists in counteracting antipsychotic-induced metabolic side effects: a systematic review. Int Clin Psychopharmacol. (2016) 31:301–6. 10.1097/YIC.0000000000000135 27294772

[176] Raskind MA Burke BL Crites NJ Tapp AM Rasmussen DD. Olanzapine-induced weight gain and increased visceral adiposity is blocked by melatonin replacement therapy in rats. Neuropsychopharmacology. (2007) 32:284–8. 10.1038/sj.npp.1301093 16710316

[177] Terron MP Delgado-Adamez J Pariente JA Barriga C Paredes SD Rodriguez AB. Melatonin reduces body weight gain and increases nocturnal activity in male wistar rats. Physiol Behav. (2013) 118:8–13. 10.1016/j.physbeh.2013.04.006 23643827

[178] Gebhardt S Theisen FM Haberhausen M Heinzel-Gutenbrunner M Wehmeier PM Krieg JC. Body weight gain induced by atypical antipsychotics: an extension of the monozygotic twin and sib pair study. J Clin Pharm Ther. (2010) 35:207–11. 10.1111/j.1365-2710.2009.01084.x 20456740

[179] Mulder H Franke B van der-Beek van der AA Arends J Wilmink FW Scheffer H. The association between HTR2C gene polymorphisms and the metabolic syndrome in patients with schizophrenia. J Clin Psychopharmacol. (2007) 27:338–43. 10.1097/JCP.0b013e3180a76dc0 17632216

[180] Ryu S Cho EY Park T Oh S Jang WS Kim SK. −759 C/T polymorphism of 5-HT2C receptor gene and early phase weight gain associated with antipsychotic drug treatment. Prog Neuropsychopharmacol Biol Psychiatry. (2007) 31:673–7. 10.1016/j.pnpbp.2006.12.021 17275977

[181] Kuzman MR Medved V Bozina N Hotujac L Sain I Bilusic H. The influence of 5-HT(2C) and MDR1 genetic polymorphisms on antipsychotic-induced weight gain in female schizophrenic patients. Psychiatry Res. (2008) 160:308–15. 10.1016/j.psychres.2007.06.006 18718676

[182] Sicard MN Zai CC Tiwari AK Souza RP Meltzer HY Lieberman JA. Polymorphisms of the HTR2C gene and antipsychotic-induced weight gain: an update and meta-analysis. Pharmacogenomics. (2010) 11:1561–71. 10.2217/pgs.10.123 21121776

[183] Kuzman MR Medved V Bozina N Grubisin J Jovanovic N Sertic J. Association study of MDR1 and 5-HT2C genetic polymorphisms and antipsychotic-induced metabolic disturbances in female patients with schizophrenia. Pharmacogenomics J. (2011) 11:35–44. 10.1038/tpj.2010.7 20195292

[184] Hong CJ Liou YJ Bai YM Chen TT Wang YC Tsai SJ. Dopamine receptor D2 gene is associated with weight gain in schizophrenic patients under long-term atypical antipsychotic treatment. Pharmacogenet Genomics. (2010) 20:359–66. 10.1097/FPC.0b013e3283397d06 20375926

[185] Lencz T Robinson DG Napolitano B Sevy S Kane JM Goldman D. DRD2 promoter region variation predicts antipsychotic-induced weight gain in first episode schizophrenia. Pharmacogenet Genomics. (2010) 20:569–72. 10.1097/FPC.0b013e32833ca24b 20664489 PMC2920359

[186] Muller DJ Zai CC Sicard M Remington E Souza RP Tiwari AK. Systematic analysis of dopamine receptor genes. (DRD1-DRD5) in antipsychotic-induced weight gain. Pharmacogenomics J. (2012) 12:156–64. 10.1038/tpj.2010.65 20714340

[187] Tybura P Trzesniowska-Drukala B Bienkowski P Beszlej A Frydecka D Mierzejewski P. Pharmacogenetics of adverse events in schizophrenia treatment: comparison study of ziprasidone, olanzapine and perazine. Psychiatry Res. (2014) 219:261–7. 10.1016/j.psychres.2014.05.039 24930580

[188] Alladi CG Mohan A Shewade DG Rajkumar RP Adithan S Subramanian K. Risperidone-induced adverse drug reactions and role of DRD2. (-141 C Ins/Del) and 5HTR2C. (-759 C>T) genetic polymorphisms in patients with schizophrenia. J Pharmacol Pharmacother. (2017) 8:28–32. 10.4103/jpp.JPP_197_16 28405133 PMC5370325

[189] Wang YC Bai YM Chen JY Lin CC Lai IC Liou YJ. Polymorphism of the adrenergic receptor alpha 2a−1291C>G genetic variation and clozapine-induced weight gain. J Neural Transm. (2005) 112:1463–8. 10.1007/s00702-005-0291-7 15795790

[190] Park YM Chung YC Lee SH Lee KJ Kim H Byun YC. Weight gain associated with the alpha2a-adrenergic receptor−1,291 C/G polymorphism and olanzapine treatment. Am J Med Genet B Neuropsychiatr Genet. (2006) 141B:394–7. 10.1002/ajmg.b.30311 16583406

[191] Sickert L Muller DJ Tiwari AK Shaikh S Zai C De Souza R. Association of the alpha 2A adrenergic receptor−1291C/G polymorphism and antipsychotic-induced weight gain in European-Americans. Pharmacogenomics. (2009) 10:1169–76. 10.2217/pgs.09.43 19604092

[192] Risselada AJ Vehof J Bruggeman R Wilffert B Cohen D Al Hadithy AF. Association between the 1291-C/G polymorphism in the adrenergic alpha-2a receptor and the metabolic syndrome. J Clin Psychopharmacol. (2010) 30:667–71. 10.1097/JCP.0b013e3181fbfac4 21105277

[193] De Luca V Souza RP Viggiano E Sickert L Teo C Zai C. Genetic interactions in the adrenergic system genes: analysis of antipsychotic-induced weight gain. Hum Psychopharmacol. (2011) 26:386–91. 10.1002/hup.1219 21823169

[194] Monteleone P Milano W Petrella C Canestrelli B Maj M. Endocannabinoid Pro129Thr FAAH functional polymorphism but not 1359G/A CNR1 polymorphism is associated with antipsychotic-induced weight gain. J Clin Psychopharmacol. (2010) 30:441–5. 10.1097/JCP.0b013e3181e742c5 20631561

[195] Tiwari AK Zai CC Likhodi O Lisker A Singh D Souza RP. A common polymorphism in the cannabinoid receptor 1. (CNR1) gene is associated with antipsychotic-induced weight gain in Schizophrenia. Neuropsychopharmacology. (2010) 35:1315–24. 10.1038/npp.2009.235 20107430 PMC3055343

[196] Park YM Choi JE Kang SG Koo SH Kim L Geum D. Cannabinoid type 1 receptor gene polymorphisms are not associated with olanzapine-induced weight gain. Hum Psychopharmacol. (2011) 26:332–7. 10.1002/hup.1210 21695734

[197] Yu W De Hert M Moons T Claes SJ Correll CU van Winkel R. CNR1 gene and risk of the metabolic syndrome in patients with schizophrenia. J Clin Psychopharmacol. (2013) 33:186–92. 10.1097/JCP.0b013e318283925e 23422373

[198] Chowdhury NI Tiwari AK Souza RP Zai CC Shaikh SA Chen S. Genetic association study between antipsychotic-induced weight gain and the melanocortin-4 receptor gene. Pharmacogenomics J. (2013) 13:272–9. 10.1038/tpj.2011.66 22310352

[199] Czerwensky F Leucht S Steimer W. MC4R rs489693: a clinical risk factor for second generation antipsychotic-related weight gain? Int J Neuropsychopharmacol. (2013) 16:2103–9. 10.1017/S1461145713000849 23920449

[200] Zhang Y Ren H Wang Q Deng W Yue W Yan H. Testing the role of genetic variation of the MC4R gene in Chinese population in antipsychotic-induced metabolic disturbance. Sci China Life Sci. (2019) 62:535–43. 10.1007/s11427-018-9489-x 30929193

[201] Czerwensky F Leucht S Steimer W. Association of the common MC4R rs17782313 polymorphism with antipsychotic-related weight gain. J Clin Psychopharmacol. (2013) 33:74–9. 10.1097/JCP.0b013e31827772db 23277235

[202] Kang SG Lee HJ Park YM Choi JE Han C Kim YK. Possible association between the−2548A/G polymorphism of the leptin gene and olanzapine-induced weight gain. Prog Neuropsychopharmacol Biol Psychiatry. (2008) 32:160–3. 10.1016/j.pnpbp.2007.08.002 17804136

[203] Yevtushenko OO Cooper SJ O'Neill R Doherty JK Woodside JV Reynolds GP. Influence of 5-HT2C receptor and leptin gene polymorphisms, smoking and drug treatment on metabolic disturbances in patients with schizophrenia. Br J Psychiatry. (2008) 192:424–8. 10.1192/bjp.bp.107.041723 18515891

[204] Gregoor JG van der Weide J Loovers HM van Megen HJ Egberts TC Heerdink ER. Association between LEP and LEPR gene polymorphisms and dyslipidemia in patients using atypical antipsychotic medication. Psychiatr Genet. (2010) 20:311–6. 10.1097/YPG.0b013e32833b6378 20562674

[205] Gregoor JG van der Weide J Loovers HM van Megen HJ Egberts TC Heerdink ER. Polymorphisms of the LEP, LEPR and HTR2C gene: obesity and BMI change in patients using antipsychotic medication in a naturalistic setting. Pharmacogenomics. (2011) 12:919–23. 10.2217/pgs.11.40 21510767

[206] Le Hellard S Theisen FM Haberhausen M Raeder MB Ferno J Gebhardt S. Association between the insulin-induced gene 2 (INSIG2) and weight gain in a German sample of antipsychotic-treated schizophrenic patients: perturbation of SREBP-controlled lipogenesis in drug-related metabolic adverse effects? Mol Psychiatry. (2009) 14:308–17. 10.1038/sj.mp.4002133 18195716

[207] Opgen-Rhein C Brandl EJ Muller DJ Neuhaus AH Tiwari AK Sander T. Association of HTR2C, but not LEP or INSIG2, genes with antipsychotic-induced weight gain in a German sample. Pharmacogenomics. (2010) 11:773–80. 10.2217/pgs.10.50 20504252

[208] Tiwari AK Zai CC Meltzer HY Lieberman JA Muller DJ Kennedy JL. Association study of polymorphisms in insulin induced gene 2 (INSIG2) with antipsychotic-induced weight gain in European and African-American schizophrenia patients. Hum Psychopharmacol. (2010) 25:253–9. 10.1002/hup.1111 20373477

[209] Liou YJ Bai YM Lin E Chen JY Chen TT Hong CJ. Gene-gene interactions of the INSIG1 and INSIG2 in metabolic syndrome in schizophrenic patients treated with atypical antipsychotics. Pharmacogenomics J. (2012) 12:54–61. 10.1038/tpj.2010.74 20877301

[210] Zhang XY Zhou DF Wu GY Cao LY Tan YL Haile CN. BDNF levels and genotype are associated with antipsychotic-induced weight gain in patients with chronic schizophrenia. Neuropsychopharmacology. (2008) 33:2200–5. 10.1038/sj.npp.1301619 17987059

[211] Tsai A Liou YJ Hong CJ Wu CL Tsai SJ Bai YM. Association study of brain-derived neurotrophic factor gene polymorphisms and body weight change in schizophrenic patients under long-term atypical antipsychotic treatment. Neuromol Med. (2011) 13:328–33. 10.1007/s12017-011-8159-5 21956459

[212] Zai GC Zai CC Chowdhury NI Tiwari AK Souza RP Lieberman JA. The role of brain-derived neurotrophic factor (BDNF) gene variants in antipsychotic response and antipsychotic-induced weight gain. Prog Neuropsychopharmacol Biol Psychiatry. (2012) 39:96–101. 10.1016/j.pnpbp.2012.05.014 22642961

[213] Zhang Y Chen M Wu Z Chen J Yu S Fang Y. Association study of Val66Met polymorphism in brain-derived neurotrophic factor gene with clozapine-induced metabolic syndrome: preliminary results. PLoS ONE. (2013) 8:e72652. 10.1371/journal.pone.0072652 23967328 PMC3742721

[214] Fonseka TM Tiwari AK Goncalves VF Lieberman JA Meltzer HY Goldstein BI. The role of genetic variation across IL-1beta, IL-2, IL-6, and BDNF in antipsychotic-induced weight gain. World J Biol Psychiatry. (2015) 16:45–56. 10.3109/15622975.2014.984631 25560300

[215] Fang H Zhen YF Liu XY Xu G Soares JC Zhao J. Association of the BDNF Val66Met polymorphism with BMI in chronic schizophrenic patients and healthy controls. Int Clin Psychopharmacol. (2016) 31:353–7. 10.1097/YIC.0000000000000142 27483421

[216] Guan F Zhang T Han W Zhu L Ni T Lin H. Relationship of SNAP25 variants with schizophrenia and antipsychotic-induced weight change in large-scale schizophrenia patients. Schizophr Res. (2020) 215:250–5. 10.1016/j.schres.2019.09.015 31653583

[217] Li N Cao T Wu X Tang M Xiang D Cai H. Progress in genetic polymorphisms related to lipid disturbances induced by atypical antipsychotic drugs. Front Pharmacol. (2019) 10:1669. 10.3389/fphar.2019.01669 32116676 PMC7011106

[218] Munafo MR Stothart G Flint J. Bias in genetic association studies and impact factor. Mol Psychiatry. (2009) 14:119–20. 10.1038/mp.2008.77 19156153

[219] Siontis KC Patsopoulos NA Ioannidis JP. Replication of past candidate loci for common diseases and phenotypes in 100 genome-wide association studies. Eur J Hum Genet. (2010) 18:832–7. 10.1038/ejhg.2010.26 20234392 PMC2987361

[220] Border R Johnson EC Evans LM Smolen A Berley N Sullivan PF. No support for historical candidate gene or candidate gene-by-interaction hypotheses for major depression across multiple large samples. Am J Psychiatry. (2019) 176:376–87. 10.1176/appi.ajp.2018.18070881 30845820 PMC6548317

[221] Arango C. Candidate gene associations studies in psychiatry: time to move forward. Eur Arch Psychiatry Clin Neurosci. (2017) 267:1–2. 10.1007/s00406-016-0765-7 28070643

[222] Malhotra AK Correll CU Chowdhury NI Muller DJ Gregersen PK Lee AT. Association between common variants near the melanocortin 4 receptor gene and severe antipsychotic drug-induced weight gain. Arch Gen Psychiatry. (2012) 69:904–12. 10.1001/archgenpsychiatry.2012.191 22566560 PMC4166499

[223] Xu B Goulding EH Zang K Cepoi D Cone RD Jones KR. Brain-derived neurotrophic factor regulates energy balance downstream of melanocortin-4 receptor. Nat Neurosci. (2003) 6:736–42. 10.1038/nn1073 12796784 PMC2710100

[224] Xu Y Jones JE Lauzon DA Anderson JG Balthasar N Heisler LK. A serotonin and melanocortin circuit mediates D-fenfluramine anorexia. J Neurosci. (2010) 30:14630–4. 10.1523/JNEUROSCI.5412-09.2010 21048120 PMC3466475

[225] Lieberman JA Stroup TS McEvoy JP Swartz MS Rosenheck RA Perkins DO. Effectiveness of antipsychotic drugs in patients with chronic schizophrenia. N Engl J Med. (2005) 353:1209–23. 10.1056/NEJMoa051688 16172203

[226] Brandl EJ Tiwari AK Zai CC Nurmi EL Chowdhury NI Arenovich T. Genome-wide association study on antipsychotic-induced weight gain in the CATIE sample. Pharmacogenomics J. (2016) 16:352–6. 10.1038/tpj.2015.59 26323598

[227] Corfitsen HT Krantz B Larsen A Drago A. Molecular pathway analysis associates alterations in obesity-related genes and antipsychotic-induced weight gain. Acta Neuropsychiatr. (2020) 32:72–83. 10.1017/neu.2019.41 31619305

[228] Yu H Wang L Lv L Ma C Du B Lu T. Genome-wide association study suggested the PTPRD polymorphisms were associated with weight gain effects of atypical antipsychotic medications. Schizophr Bull. (2016) 42:814–23. 10.1093/schbul/sbv179 26656879 PMC4838100

[229] Uetani N Kato K Ogura H Mizuno K Kawano K Mikoshiba K. Impaired learning with enhanced hippocampal long-term potentiation in PTPdelta-deficient mice. EMBO J. (2000) 19:2775–85. 10.1093/emboj/19.12.2775 10856223 PMC203365

[230] Maciukiewicz M Gorbovskaya I Tiwari AK Zai CC Freeman N Meltzer HY. Genetic validation study of protein tyrosine phosphatase receptor type D (PTPRD) gene variants and risk for antipsychotic-induced weight gain. J Neural Transm. (2019) 126:27–33. 10.1007/s00702-018-1921-1 30229349

[231] Maciukiewicz M Tiwari AK Zai CC Gorbovskaya I Laughlin CP Nurmi EL. Genome-wide association study on antipsychotic-induced weight gain in Europeans and African-Americans. Schizophr Res. (2019) 212:204–12. 10.1016/j.schres.2019.07.022 31447353

[232] Goodarzi MO Guo X Cui J Jones MR Haritunians T Xiang AH. Systematic evaluation of validated type 2 diabetes and glycaemic trait loci for association with insulin clearance. Diabetologia. (2013) 56:1282–90. 10.1007/s00125-013-2880-6 23494448 PMC3651757

[233] Keaton JM Hellwege JN Ng MCY Palmer ND Pankow JS Fornage M. Genome-wide interaction with selected type 2 diabetes loci reveals novel loci for type 2 diabetes in African Americans. Pac Symp Biocomput. (2017) 22:242–53. 10.1142/9789813207813_0024 27896979 PMC5146756

[234] Zhang L Dai Y Bian L Wang W Wang W Muramatsu M. Association of the cell death-inducing DNA fragmentation factor alpha-like effector A (CIDEA) gene V115F (G/T) polymorphism with phenotypes of metabolic syndrome in a Chinese population. Diabetes Res Clin Pract. (2011) 91:233–8. 10.1016/j.diabres.2010.10.016 21106268

[235] Wu J Zhang L Zhang J Dai Y Bian L Song M. The genetic contribution of CIDEA polymorphisms, haplotypes and loci interaction to obesity in a Han Chinese population. Mol Biol Rep. (2013) 40:5691–9. 10.1007/s11033-013-2671-7 24057179

[236] Ter Hark SE Jamain S Schijven D Lin BD Bakker MK Boland-Auge A. A new genetic locus for antipsychotic-induced weight gain: a genome-wide study of first-episode psychosis patients using amisulpride (from the OPTiMiSE cohort). J Psychopharmacol. (2020) 34:524–31. 10.1177/0269881120907972 32126890 PMC7222287

[237] Leucht S Winter-van Rossum I Heres S Arango C Fleischhacker WW Glenthoj B. The optimization of treatment and management of schizophrenia in Europe (OPTiMiSE) trial: rationale for its methodology and a review of the effectiveness of switching antipsychotics. Schizophr Bull. (2015) 41:549–58. 10.1093/schbul/sbv019 25786408 PMC4393704

[238] Gao L Zheng Z Cao L Shen S Yang Y Zhao Z. The growth hormone receptor (GHR) exon 3 polymorphism and its correlation with metabolic profiles in obese Chinese children. Pediatr Diabetes. (2011) 12 (4 Pt. 2):429–34. 10.1111/j.1399-5448.2010.00747.x 21470351

[239] Hellwege JN Palmer ND Ziegler JT Langefeld CD Lorenzo C Norris JM. *Gene*tic variants in selenoprotein P plasma 1 gene (SEPP1) are associated with fasting insulin and first phase insulin response in hispanics. Gene. (2014) 534:33–9. 10.1016/j.gene.2013.10.035 24161883 PMC3856675

[240] Chen M Liu B Wilkinson D Hutchison AT Thompson CH Wittert GA. Selenoprotein P is elevated in individuals with obesity, but is not independently associated with insulin resistance. Obes Res Clin Pract. (2017) 11:227–32. 10.1016/j.orcp.2016.07.004 27524654

[241] Gharipour M Sadeghi M Salehi M Behmanesh M Khosravi E Dianatkhah M. Association of expression of selenoprotein P in mRNA and protein levels with metabolic syndrome in subjects with cardiovascular disease: results of the Selenegene study. J Gene Med. (2017) 19:e2945. 10.1002/jgm.2945 28190280

[242] Akbaba G Akbaba E Sahin C Kara M. The relationship between gestational diabetes mellitus and selenoprotein-P plasma 1 (SEPP1) gene polymorphisms. Gynecol Endocrinol. (2018) 34:849–52. 10.1080/09513590.2018.1460659 29648467

[243] Espinosa E Salame L Marrero-Rodriguez D Romero-Nieves AM Cuenca D Castelan-Martinez OD. Expression of the growth hormone receptor isoforms and its correlation with the metabolic profile in morbidly obese subjects. Endocrine. (2019) 63:573–81. 10.1007/s12020-018-1794-y 30361972

[244] Vock C Doring F Nitz I. Transcriptional regulation of HMG-CoA synthase and HMG-CoA reductase genes by human ACBP. Cell Physiol Biochem. (2008) 22:515–24. 10.1159/000185525 19088433

[245] Bahr SM Weidemann BJ Castro AN Walsh JW deLeon O Burnett CM. Risperidone-induced weight gain is mediated through shifts in the gut microbiome and suppression of energy expenditure. EBioMedicine. (2015) 2:1725–34. 10.1016/j.ebiom.2015.10.018 26870798 PMC4740326

[246] Kao AC Spitzer S Anthony DC Lennox B Burnet PWJ. Prebiotic attenuation of olanzapine-induced weight gain in rats: analysis of central and peripheral biomarkers and gut microbiota. Transl Psychiatry. (2018) 8:66. 10.1038/s41398-018-0116-8 29540664 PMC5852210

[247] Dhaliwal N Dhaliwal J Singh DP Kondepudi KK Bishnoi M Chopra K. The probiotic mixture VSL#3 reverses olanzapine-induced metabolic dysfunction in mice. Methods Mol Biol. (2019) 2011:531–44. 10.1007/978-1-4939-9554-7_31 31273720

[248] Skonieczna-Zydecka K Loniewski I Misera A Stachowska E Maciejewska D Marlicz W. Second-generation antipsychotics and metabolism alterations: a systematic review of the role of the gut microbiome. Psychopharmacology. (2019) 236:1491–512. 10.1007/s00213-018-5102-6 30460516 PMC6598971

[249] Davey KJ O'Mahony SM Schellekens H O'Sullivan O Bienenstock J Cotter PD. Gender-dependent consequences of chronic olanzapine in the rat: effects on body weight, inflammatory, metabolic and microbiota parameters. Psychopharmacology. (2012) 221:155–69. 10.1007/s00213-011-2555-2 22234378

[250] Davey KJ Cotter PD O'Sullivan O Crispie F Dinan TG Cryan JF. Antipsychotics and the gut microbiome: olanzapine-induced metabolic dysfunction is attenuated by antibiotic administration in the rat. Transl Psychiatry. (2013) 3:e309. 10.1038/tp.2013.83 24084940 PMC3818006

[251] Morgan AP Crowley JJ Nonneman RJ Quackenbush CR Miller CN Ryan AK. The antipsychotic olanzapine interacts with the gut microbiome to cause weight gain in mouse. PLoS ONE. (2014) 9:e115225. 10.1371/journal.pone.0115225 25506936 PMC4266663

[252] Bahr SM Tyler BC Wooldridge N Butcher BD Burns TL Teesch LM. Use of the second-generation antipsychotic, risperidone, and secondary weight gain are associated with an altered gut microbiota in children. Transl Psychiatry. (2015) 5:e652. 10.1038/tp.2015.135 26440540 PMC4930121

[253] Indiani C Rizzardi KF Castelo PM Ferraz LFC Darrieux M Parisotto TM. Childhood obesity and firmicutes/bacteroidetes ratio in the gut microbiota: a systematic review. Child Obes. (2018) 14:501–9. 10.1089/chi.2018.0040 30183336

[254] Castaner O Goday A Park YM Lee SH Magkos F Shiow STE. The gut microbiome profile in obesity: a systematic review. Int J Endocrinol. (2018) 2018:4095789. 10.1155/2018/4095789 29849617 PMC5933040

[255] Turnbaugh PJ Hamady M Yatsunenko T Cantarel BL Duncan A Ley RE. A core gut microbiome in obese and lean twins. Nature. (2009) 457:480–4. 10.1038/nature07540 19043404 PMC2677729

[256] Ravussin Y Koren O Spor A LeDuc C Gutman R Stombaugh J. Responses of gut microbiota to diet composition and weight loss in lean and obese mice. Obesity. (2012) 20:738–47. 10.1038/oby.2011.111 21593810 PMC3871199

[257] Turnbaugh PJ Backhed F Fulton L Gordon JI. Diet-induced obesity is linked to marked but reversible alterations in the mouse distal gut microbiome. Cell Host Microbe. (2008) 3:213–23. 10.1016/j.chom.2008.02.015 18407065 PMC3687783

[258] Ridaura VK Faith JJ Rey FE Cheng J Duncan AE Kau AL. Gut microbiota from twins discordant for obesity modulate metabolism in mice. Science. (2013) 341:1241214. 10.1126/science.1241214 24009397 PMC3829625

[259] Borgo F Verduci E Riva A Lassandro C Riva E Morace G. Relative abundance in bacterial and fungal gut microbes in obese children: a case control study. Child Obes. (2017) 13:78–84. 10.1089/chi.2015.0194 27007700

[260] Spor A Koren O Ley R. Unravelling the effects of the environment and host genotype on the gut microbiome. Nat Rev Microbiol. (2011) 9:279–90. 10.1038/nrmicro2540 21407244

[261] Yatsunenko T Rey FE Manary MJ Trehan I Dominguez-Bello MG Contreras M. Human gut microbiome viewed across age and geography. Nature. (2012) 486:222–7. 10.1038/nature11053 22699611 PMC3376388

[262] Belkaid Y Hand TW. Role of the microbiota in immunity and inflammation. Cell. (2014) 157:121–41. 10.1016/j.cell.2014.03.011 24679531 PMC4056765

[263] Flowers SA Evans SJ Ward KM McInnis MG Ellingrod VL. Interaction between atypical antipsychotics and the gut microbiome in a bipolar disease cohort. Pharmacotherapy. (2017) 37:261–7. 10.1002/phar.1890 28035686

[264] Correll CU Sikich L Reeves G Johnson J Keeton C Spanos M. Metformin add-on vs. antipsychotic switch vs continued antipsychotic treatment plus healthy lifestyle education in overweight or obese youth with severe mental illness: results from the IMPACT trial. World Psychiatry. (2020) 19:69–80. 10.1002/wps.20714 31922663 PMC6953545

[265] Detke HC DelBello MP Landry J Hoffmann VP Heinloth A Dittmann RW. A 52-week study of olanzapine with a randomized behavioral weight counseling intervention in adolescents with schizophrenia or bipolar I disorder. J Child Adolesc Psychopharmacol. (2016) 26:922–34. 10.1089/cap.2016.0010 27676420

[266] Nicol GE Kolko R Lenze EJ Yingling MD Miller JP Ricchio AR. Adiposity, hepatic triglyceride, and carotid intima media thickness during behavioral weight loss treatment in antipsychotic-treated youth: a randomized pilot study. J Child Adolesc Psychopharmacol. (2019) 29:439–47. 10.1089/cap.2018.0120 30994376 PMC6661918

[267] Rice J Ramtekkar U. Integrative management of metabolic syndrome in youth prescribed second-generation antipsychotics. Med Sci. (2020) 8:34. 10.3390/medsci8030034 32824428 PMC7564042

[268] Vancampfort D Firth J Correll CU Solmi M Siskind D De Hert M. The impact of pharmacological and non-pharmacological interventions to improve physical health outcomes in people with schizophrenia: a meta-review of meta-analyses of randomized controlled trials. World Psychiatry. (2019) 18:53–66. 10.1002/wps.20614 30600626 PMC6313230

[269] Turnbaugh PJ Ley RE Mahowald MA Magrini V Mardis ER Gordon JI. An obesity-associated gut microbiome with increased capacity for energy harvest. Nature. (2006) 444:1027–31. 10.1038/nature05414 17183312

[270] Ferrer M Ruiz A Lanza F Haange SB Oberbach A Till H. Microbiota from the distal guts of lean and obese adolescents exhibit partial functional redundancy besides clear differences in community structure. Environ Microbiol. (2013) 15:211–26. 10.1111/j.1462-2920.2012.02845.x 22891823

[271] Hume MP Nicolucci AC Reimer RA. Prebiotic supplementation improves appetite control in children with overweight and obesity: a randomized controlled trial. Am J Clin Nutr. (2017) 105:790–9. 10.3945/ajcn.116.140947 28228425

[272] Dickerson FB Stallings C Origoni A Katsafanas E Savage CL Schweinfurth LA. Effect of probiotic supplementation on schizophrenia symptoms and association with gastrointestinal functioning: a randomized, placebo-controlled trial. Prim Care Companion CNS Disord. (2014) 15. 10.4088/PCC.13m01579 24940526 PMC4048142

[273] Tomasik J Yolken RH Bahn S Dickerson FB. Immunomodulatory effects of probiotic supplementation in schizophrenia patients: a randomized, placebo-controlled trial. Biomark Insights. (2015) 10:47–54. 10.4137/BMI.S22007 26052224 PMC4454091

[274] Townsend LK Peppler WT Bush ND Wright DC. Obesity exacerbates the acute metabolic side effects of olanzapine. Psychoneuroendocrinology. (2018) 88:121–8. 10.1016/j.psyneuen.2017.12.004 29241148

[275] Nurmi EL. Do Microbiome-Bile Acid Interactions Explain Antipsychotic-Induced Weight Gain. Los Angeles, CA: American Academy of Child and Adolescent Psychiatry (2018).

[276] Nurmi EL. Altered Bile Acid Signaling Correlates With Antipsychotic-Induced Weight Gain in Children. Los Angeles, CA: Society of Biological Psychiatry (2019).

[277] Sun L Xie C Wang G Wu Y Wu Q Wang X. Gut microbiota and intestinal FXR mediate the clinical benefits of metformin. Nat Med. (2018) 24:1919–29. 10.1038/s41591-018-0222-4 30397356 PMC6479226

[278] Marteene W Winckel K Hollingworth S Kisely S Gallagher E Hahn M. Strategies to counter antipsychotic-associated weight gain in patients with schizophrenia. Expert Opin Drug Saf . (2019) 18:1149–60. 10.1080/14740338.2019.1674809 31564170

[279] Ojala K Repo-Tiihonen E Tiihonen J Niskanen L. Statins are effective in treating dyslipidemia among psychiatric patients using second-generation antipsychotic agents. J Psychopharmacol. (2008) 22:33–8. 10.1177/0269881107077815 17715204

[280] Malin SK Kashyap SR. Effects of metformin on weight loss: potential mechanisms. Curr Opin Endocrinol Diabetes Obes. (2014) 21:323–9. 10.1097/MED.0000000000000095 25105996

[281] Mizuno Y Suzuki T Nakagawa A Yoshida K Mimura M Fleischhacker WW. Pharmacological strategies to counteract antipsychotic-induced weight gain and metabolic adverse effects in schizophrenia: a systematic review and meta-analysis. Schizophr Bull. (2014) 40:1385–403. 10.1093/schbul/sbu030 24636967 PMC4193713

[282] Anagnostou E Aman MG Handen BL Sanders KB Shui A Hollway JA. Metformin for treatment of overweight induced by atypical antipsychotic medication in young people with autism spectrum disorder: a randomized clinical trial. JAMA Psychiatry. (2016) 73:928–37. 10.1001/jamapsychiatry.2016.1232 27556593

[283] Shin L Bregman H Breeze JL Noyes N Frazier JA. Metformin for weight control in pediatric patients on atypical antipsychotic medication. J Child Adolesc Psychopharmacol. (2009) 19:275–9. 10.1089/cap.2008.094 19519262 PMC6469519

[284] Morrison JA Cottingham EM Barton BA. Metformin for weight loss in pediatric patients taking psychotropic drugs. Am J Psychiatry. (2002) 159:655–7. 10.1176/appi.ajp.159.4.655 11925306

[285] Handen BL Anagnostou E Aman MG Sanders KB Chan J Hollway JA. A randomized, placebo-controlled trial of metformin for the treatment of overweight induced by antipsychotic medication in young people with autism spectrum disorder: open-label extension. J Am Acad Child Adolesc Psychiatry. (2017) 56:849–56.e846. 10.1016/j.jaac.2017.07.790 28942807

[286] Arman S Sadramely MR Nadi M Koleini N. A randomized, double-blind, placebo-controlled trial of metformin treatment for weight gain associated with initiation of risperidone in children and adolescents. Saudi Med J. (2008) 29:1130–4. 18690305

[287] Maayan L Vakhrusheva J Correll CU. Effectiveness of medications used to attenuate antipsychotic-related weight gain and metabolic abnormalities: a systematic review and meta-analysis. Neuropsychopharmacology. (2010) 35:1520–30. 10.1038/npp.2010.21 20336059 PMC3055458

[288] Hendrick V Dasher R Gitlin M Parsi M. Minimizing weight gain for patients taking antipsychotic medications: the potential role for early use of metformin. Ann Clin Psychiatry. (2017) 29:120–4. 28463344

[289] Ebdrup BH Knop FK Ishoy PL Rostrup E Fagerlund B Lublin H. Glucagon-like peptide-1 analogs against antipsychotic-induced weight gain: potential physiological benefits. BMC Med. (2012) 10:92. 10.1186/1741-7015-10-92 22891821 PMC3573939

[290] Smith GC Zhang ZY Mulvey T Petersen N Lach S Xiu P. Clozapine directly increases insulin and glucagon secretion from islets: implications for impairment of glucose tolerance. Schizophr Res. (2014) 157:128–33. 10.1016/j.schres.2014.05.003 24906220

[291] Teff KL Rickels MR Grudziak J Fuller C Nguyen HL Rickels K. Antipsychotic-induced insulin resistance and postprandial hormonal dysregulation independent of weight gain or psychiatric disease. Diabetes. (2013) 62:3232–40. 10.2337/db13-0430 23835329 PMC3749337

[292] Siskind D Hahn M Correll CU Fink-Jensen A Russell AW Bak N. Glucagon-like peptide-1 receptor agonists for antipsychotic-associated cardio-metabolic risk factors: a systematic review and individual participant data meta-analysis. Diabetes Obes Metab. (2019) 21:293–302. 10.1111/dom.13522 30187620

[293] Kelly AS. Glucagon-like peptide-1 receptor agonist treatment for pediatric obesity. Endocr Dev. (2016) 30:23–8. 10.1159/000439323 26683061

[294] Poyurovsky M Fuchs C Pashinian A Levi A Weizman R Weizman A. Reducing antipsychotic-induced weight gain in schizophrenia: a double-blind placebo-controlled study of reboxetine-betahistine combination. Psychopharmacology. (2013) 226:615–22. 10.1007/s00213-012-2935-2 23239133

[295] Kang D Jing Z Li R Hei G Shao T Li L. Effect of betahistine and metformin on antipsychotic-induced weight gain: an analysis of two clinical trials. Front Psychiatry. (2018) 9:620. 10.3389/fpsyt.2018.00620 30542300 PMC6277778

[296] Smith RC Maayan L Wu R Youssef M Jing Z Sershen H. Betahistine effects on weight-related measures in patients treated with antipsychotic medications: a double-blind placebo-controlled study. Psychopharmacology. (2018) 235:3545–58. 10.1007/s00213-018-5079-1 30382354

[297] Deng C Lian J Pai N Huang XF. Reducing olanzapine-induced weight gain side effect by using betahistine: a study in the rat model. J Psychopharmacol. (2012) 26:1271–9. 10.1177/0269881112449396 22695490

[298] Lian J Huang XF Pai N Deng C. Effects of olanzapine and betahistine co-treatment on serotonin transporter, 5-HT2A and dopamine D2 receptor binding density. Prog Neuropsychopharmacol Biol Psychiatry. (2013) 47:62–8. 10.1016/j.pnpbp.2013.08.005 23994047

[299] Lian J Huang XF Pai N Deng C. Preventing olanzapine-induced weight gain using betahistine: a study in a rat model with chronic olanzapine treatment. PLoS ONE. (2014) 9:e104160. 10.1371/journal.pone.0104160 25084453 PMC4118967

[300] Lian J Huang XF Pai N Deng C. Betahistine ameliorates olanzapine-induced weight gain through modulation of histaminergic, NPY and AMPK pathways. Psychoneuroendocrinology. (2014) 48:77–86. 10.1016/j.psyneuen.2014.06.010 24992721

[301] Lian J Huang XF Pai N Deng C. Chronic betahistine co-treatment reverses olanzapine's effects on dopamine D(2) but not 5-HT2A/2C bindings in rat brains. Prog Neuropsychopharmacol Biol Psychiatry. (2015) 56:75–80. 10.1016/j.pnpbp.2014.08.006 25149912

[302] Lian J Huang XF Pai N Deng C. Ameliorating antipsychotic-induced weight gain by betahistine: mechanisms and clinical implications. Pharmacol Res. (2016) 106:51–63. 10.1016/j.phrs.2016.02.011 26892184

[303] Goh KK Chen CH Lu ML. Topiramate mitigates weight gain in antipsychotic-treated patients with schizophrenia: meta-analysis of randomised controlled trials. Int J Psychiatry Clin Pract. (2019) 23:14–32. 10.1080/13651501.2018.1449864 29557263

[304] Shapiro M Reid A Olsen B Taasan M McNamara J Nguyen M. Topiramate, zonisamide and weight loss in children and adolescents prescribed psychiatric medications: a medical record review. Int J Psychiatry Med. (2016) 51:56–68. 10.1177/0091217415621266 26681236

[305] Cipolla-Neto J Amaral FG Afeche SC Tan DX Reiter RJ. Melatonin, energy metabolism, and obesity: a review. J Pineal Res. (2014) 56:371–81. 10.1111/jpi.12137 24654916

[306] Mostafavi A Solhi M Mohammadi MR Hamedi M Keshavarzi M Akhondzadeh S. Melatonin decreases olanzapine induced metabolic side-effects in adolescents with bipolar disorder: a randomized double-blind placebo-controlled trial. Acta Med Iran. (2014) 52:734–9. 25369006

[307] Mostafavi SA Solhi M Mohammadi MR Akhondzadeh S. Melatonin for reducing weight gain following administration of atypical antipsychotic olanzapine for adolescents with bipolar disorder: a randomized, double-blind, placebo-controlled trial. J Child Adolesc Psychopharmacol. (2017) 27:440–4. 10.1089/cap.2016.0046 28339282

[308] Cajochen C Krauchi K Wirz-Justice A. Role of melatonin in the regulation of human circadian rhythms and sleep. J Neuroendocrinol. (2003) 15:432–7. 10.1046/j.1365-2826.2003.00989.x 12622846

[309] Fatima Y Doi SA Mamun AA. Sleep quality and obesity in young subjects: a meta-analysis. Obes Rev. (2016) 17:1154–66. 10.1111/obr.12444 27417913

[310] Gao K Fang F Wang Z Calabrese JR. Subjective versus objective weight gain during acute treatment with second-generation antipsychotics in schizophrenia and bipolar disorder. J Clin Psychopharmacol. (2016) 36:637–42. 10.1097/JCP.0000000000000596 27753728

[311] Mangurian C Newcomer JW Modlin C Schillinger D. Diabetes and cardiovascular care among people with severe mental illness: a literature review. J Gen Intern Med. (2016) 31:1083–91. 10.1007/s11606-016-3712-4 27149967 PMC4978675

[312] Mitchell AJ Vancampfort D Sweers K van Winkel R Yu W De Hert M. Prevalence of metabolic syndrome and metabolic abnormalities in schizophrenia and related disorders–a systematic review and meta-analysis. Schizophr Bull. (2013) 39:306–18. 10.1093/schbul/sbr148 22207632 PMC3576174

[313] Casey DE Carson WH Saha AR Liebeskind A Ali MW Jody D. Switching patients to aripiprazole from other antipsychotic agents: a multicenter randomized study. Psychopharmacology. (2003) 166:391–9. 10.1007/s00213-002-1344-3 12610718

[314] Weiden PJ Simpson GM Potkin SG O'Sullivan RL. Effectiveness of switching to ziprasidone for stable but symptomatic outpatients with schizophrenia. J Clin Psychiatry. (2003) 64:580–8. 10.4088/JCP.v64n0514 12755663

[315] Tchang BG Abel B Zecca C Saunders KH Shukla AP. An up-to-date evaluation of lorcaserin hydrochloride for the treatment of obesity. Expert Opin Pharmacother. (2020) 21:21–8. 10.1080/14656566.2019.1685496 31693425

[316] O'ConnorEAEvansCVBurdaBUWalshESEderMLozanoP. Screening for obesity and intervention for weight management in children and adolescents: evidence report and systematic review for the US preventive services task force. JAMA. (2017) 317:2427–44. 10.1001/jama.2017.033228632873

[317] Kawata Y Okuda S Hotta N Igawa H Takahashi M Ikoma M. A novel and selective melanin-concentrating hormone receptor 1 antagonist ameliorates obesity and hepatic steatosis in diet-induced obese rodent models. Eur J Pharmacol. (2017) 796:45–53. 10.1016/j.ejphar.2016.12.018 27986627

[318] Saunders KH Igel LI Aronne LJ. An update on naltrexone/bupropion extended-release in the treatment of obesity. Expert Opin Pharmacother. (2016) 17:2235–42. 10.1080/14656566.2016.1244527 27700187

